# SARS-CoV-2-induced damage to rat cortical neuronal networks *ex vivo* is mediated by the pro-inflammatory activation of the cGAS-STING pathway

**DOI:** 10.1007/s13365-025-01283-6

**Published:** 2025-10-25

**Authors:** Pamela Martinez-Orellana, Matteo Manzati, Diletta Pozzi, Yingying Xiao, Alessio Di Clemente, Marika Mearelli, Chiara Kalebić, Valentina Perrera, Denise Ferrarini, Tea Carletti, Carmen Falcone, Michele Giugliano, Alessandro Marcello

**Affiliations:** 1 The International Centre for Genetic Engineering and Biotechnology (ICGEB), Padriciano 99, 34149 Trieste, Italy; 2 International School for Advances Studies (SISSA), Via Bonomea 265, 34136 Trieste, Italy; 3 Universita Degli Studi Di Modena E Reggio Emilia, Via Campi 287, 41125 Modena, Italy; 4 Achucarro Basque Center for Neuroscience, 48940 Leioa, Basque Country Spain; 5 AREA Science Park, Padriciano 99, 34149 Trieste, Italy; 6 National Interuniv. Consortium of Materials Science and Technology (INSTM), Florence, Italy

**Keywords:** SARS-CoV-2, cGAS, glia, DDR, MEA

## Abstract

Severe Acute Respiratory Syndrome Coronavirus-2 infection has been associated to neurological symptoms characteristic of long-lasting post-acute coronavirus disease. However, the complex mechanisms involved in these clinical manifestations are still unclear. Glial cells are key to inflammation and neurodegeneration in response to central nervous system infection. To investigate this pathway, induced pluripotent stem cells human astrocytes and human microglial HMC3 cells were infected with SARS-CoV-2. Astrocytes showed to be prone to infection, while HMC3 supported only marginal virus replication. A significant IFN-β response was induced in astrocytes, while both cell types showed some level of chemoattractant production. Interestingly, both glial cells showed signs of senescence and activation of the pro-inflammatory cGAS-STING pathway. To investigate if glial cells infection could impair the function of neuronal networks, primary rat cortical cultures seeded on multi-electrode arrays were used to monitor the electrical activity after exposure to SARS-CoV-2. Effective SARS-CoV-2 infection of the glia led to a major loss of synaptic connections, an increase expression and production of pro-inflammatory cytokines and chemokines, and an increase of DNA damage foci. Intriguingly, the pro-inflammatory response was cGAS-STING dependent. Finally, an antagonist of the cGAS-STING pathway was able to ameliorate the decrease in electrical activity early post-infection. These data point to SARS-CoV-2 infection of the glia as a culprit for neurological complications during COVID-19.

**Supplementary Information:**

The online version contains supplementary material available at 10.1007/s13365-025-01283-6.

## Introduction

The Severe Acute Respiratory Syndrome Coronavirus-2 (SARS-CoV-2), responsible for the Coronavirus disease 2019 (COVID-19) pandemic (Zhu et al. 2020), has been associated not only to common respiratory symptoms, but also to neurological manifestations both in severe and mildly affected COVID-19 patients (Monje and Iwasaki 2022). At least a third of convalescent individuals exhibited neuropsychiatric and neurological symptoms, which increased in the more severe cases of infection (Mao et al. 2020). In autopsies performed on COVID-19 patients, signs of histopathological damage were found in the brain, together with an altered cerebral cortical thickness; these examinations revealed that astrocytes were infected with the virus (Crunfli et al. 2022). Additional brain autopsies of deceased patients demonstrated the presence of SARS-CoV-2 in cortical neurons, together with infiltrates of immune cells (Song et al. 2021). SARS-CoV-2 RNA and nucleocapsid protein (N) have been observed also in the hypothalamus, cerebellum, cervical spinal cord and basal ganglia with a pattern consistent with neuronal staining (Stein et al. 2022). The presence of the virus has also been reported in the cerebrospinal fluid and in the neuronal cells of COVID-19 patients (Virhammar et al. 2020). Notwithstanding the early evidence of SARS-CoV-2 infection of the central nervous system (CNS), recent data challenged this notion. The work by Radke et al. (Radke et al. 2024) claim that the neurocognitive symptoms observed in patients in the acute and late disease phase of COVID-19 are caused by a local CNS reaction to systemic infection instead of a direct SARS-CoV-2 infection of the brain. They used validated antibodies (Krasemann et al. 2022) for in situ immunostaining of autoptic tissue and could not find viral antigen arguing lack of direct infection of the central nervous tissues.

SARS-CoV-2 binds to the angiotensin-converting enzyme 2 (ACE2) host-cell receptor through the surface protein spike (S) to enter the cell (Hoffmann et al. 2020). According to transcriptomics, ACE2 seems to be expressed in almost all brain regions, yet mostly limited to neurons, astrocytes and other glial cells (Chen and Li 2021; Haverty et al. 2024; Wälchli et al. 2024). Initially, olfactory neurons were considered as a candidate for the main entry point of the virus into the brain (Jiao et al. 2020; Meinhardt et al. 2021), but later studies questioned this hypothesis (Butowt et al. 2021). Abnormalities in the frontal electroencephalogram pattern detected in patients with COVID-19-related encephalopathy (Antony and Haneef 2020) and foci of histopathological damage in the brain (Song et al. 2021) led to more attention on the direct infection of the CNS. Moreover, SARS-CoV-2 was also found to damage the blood–brain barrier, causing the loss of its protective role (Pellegrini et al. 2020).

A fundamental yet poorly explored question is to understand whether the damage induced in neurons and glia by SARS-CoV-2 results from direct infection, or to the damage mediated by the local immune response and subsequent inflammation. COVID-19 patients develop an exaggerated pro-inflammatory response to SARS-CoV-2 (Fara et al. 2020; Darif et al. 2021), the so called “cytokine storm”, or cytokine release syndrome, which contributes greatly to tissue damage principally in the lungs (Low et al. 2023). The cGAS-STING pathway is a key mediator of the inflammatory responses following infection, cellular stress and tissue damage (Decout et al. 2021). We recently demonstrated that SARS-CoV-2 infection compromises genome integrity by causing virus-induced DNA damage and senescence leading to the activation of pro-inflammatory pathways, including cGAS-STING (Gioia et al. 2023). While these studies were performed in lung and nasal epithelial cells, the same mechanisms could apply also in the central nervous system where astrocytes are the main site of SARS-CoV-2 infection (Andrews et al. 2022; Crunfli et al. 2022). High levels of biomarkers for CNS lesions, such as glial fibrillary acid protein (GFAP) and neurofilament light chain protein (NfL), were found in the cerebrospinal fluid of severe and moderate cases of COVID-19 (Virhammar et al. 2020; Kanberg et al. 2021), suggesting that the astrocytes of these patients were in an active state. Microglia, which is the main cell type active in the CNS during a viral infection with the function to recruit and activate peripheral monocytes/macrophages and specific viral T-cells, has also been shown to cause neurotoxicity (Tremblay et al. 2011). Microglia activation following infection leads to synaptic loss (Wake et al. 2009) and contributes with astrocytes to the release of several cytokines such as IL-6, IL-10, TNFs, IL-1β as well as chemokines and colony-stimulating factors capable of increasing the permeability of the blood brain barrier (Sun et al. 2020). Cytokines can cause aberrant neuronal excitability by affecting neurotransmitter release, cell survival, and synaptic integrity leading to functional abnormalities.

In this work, we investigated the effect of SARS-CoV-2 infection on human glial cells and rat cortical neurons. Glial cells display patterns of virus-induced DNA damage, cellular senescence, and activation of pro-inflammatory responses. Therefore, we hypothesized that these cells could be responsible for the inflammatory cascades in the brain. We demonstrated that wild type primary rat neurons and glial cells are susceptible to SARS-CoV-2 infection, thus providing an amenable model for brain infection ex vivo. We indeed confirmed that their network-level electrophysiological phenotype—characterized by multi-electrode arrays (MEAs) extracellular voltage recordings – is altered with irreversible signal loss. The induction of pro-inflammatory cytokines and chemokines arising from SARS-CoV-2 infection was also observed. We then focused on the cGAS–STING pathway showing that it is induced by infection of astrocytes and microglia, and its inhibition partially rescues the electrical activity dampened by SARS-CoV-2 in rat cortical cells.

## Materials and methods

### Cells

Human induced pluripotent stem cells (iPSC) were used to produce astrocytes, as already reported in Padmashri et al. (Padmashri et al. 2021). Briefly, FX11-9u hiPSC line (RRID: CVCL_EJ77) from WiCell were cultured in mTeSRTM Plus (STEMCELL Technologies, 05825) medium until developing into embryoid bodies. Embryoid bodies were generated by splitting the iPSC manually and from the embryoid bodies neural progenitor cells (NPCs) were obtained. NPCs were then manually isolated and allowed to expand in neurospheres. To differentiate astrocytes, neurospheres were dissociated into single cells and were cultured with a specific astrocyte dissociation medium: DME/F12, 1 × N2 supplement, 1 × B27-RA supplement, BMP4 (10 ng/mL, Peprotech, 120-05ET), and FGF-basic (20 ng/mL). Then, cells were seeded at a density of 26,000 cells/cm^2^ per well into 24 well plate or 10,000 cells/cm^2^ per well into 6 well plate.

HMC3 cells (ATCC®CRL-3304) were grown according to the instructions provided in the data sheet. The cell culture medium used was prepared with Eagle’s Minimum Essential Medium (EMEM, ATCC® 302,003™) supplemented with 10% fetal bovine serum (FBS) (Euroclone, ECS500L), and gentamicin (50 μg/ml, Gibco, 15,710–049). Cells were seeded in 24 well plate at a density of 15,000cells/cm^2^ cells per well, in 12 well plates at a density of 11,000 cells/cm^2^ per well and in 6 well plates at a density of 10,000 cells/cm^2^ per well.

Primary cultures of cortical neurons were prepared by enzymatically and mechanically dissociating brain tissue of P0-2 Wistar rats, as already described for MEA seeding (Manzati et al. 2020). All procedures were performed in accordance with the guidelines of the Italian Animal Welfare Act and their use was approved by the Local Veterinary Service, the SISSA Ethics Committee board, and the National Ministry of Health, in accordance with the European Union guidelines for animal care (Directive 2010/63/EU). For electrophysiology, cells were plated at a density of 6,500 cells/mm2 on microelectrodes arrays (MEAs; 120MEA100/30iR-Ti-gr, MultiChannel Systems, Reutlingen, Germany). For immunohistochemical and biochemical analysis, cells were seeded on glass coverslips at a density of 750 or 1500 cells/mm^2^. Before plating, the surface of each MEA and glass coverslip was treated overnight at 37 °C with poly(ethyleneimine) (PEI; Merck KGaA, Darmstadt, P3143) 0.1% in milliQ water to enhance cell adhesion. After plating, cells were kept in a humidified environment of 95% air-5% CO2 at 37ºC in a cell culture medium consisting of MEM (Gibco, 21,090–022), supplemented with 5% (v/v) heat-inactivated horse serum (Merck KGaA, Darmstadt, H1138,), 20 mM glucose (Merck KGaA, Darmstadt, 1.04074.1000,), 0.1% (v/v) gentamycin (Merck KGaA, Darmstadt, G1397), and 100 µM GlutaMAX (ThermoFisher Scientific, Waltham, Massachusetts, 35,050,061). Every two days, half the volume of cell medium was replaced with fresh media. After one week in culture GlutaMax concentration was reduced to 50 µM.

### SARS-CoV-2 propagation and in vitro or ex vivo infection

Vero E6 cells (ATCC-1586) were cultured in Dulbecco’s modified Eagle’s medium (DMEM, Gibco, 31,885–023) supplemented with 10% fetal bovine serum (Euroclone, ECS500L) and 50 µg/mL gentamicin (Gibco, 15,710–049). Cell cultures were maintained at 37 °C, 5% CO2. Cells were routinely tested for mycoplasma contamination. Working stocks of ancestral SARS-CoV-2 ICGEB-FVG_5 isolated in Trieste, Italy (Licastro et al. 2020) were propagated six times on semiconfluent Vero E6 cells. The virus stock was then enriched using Intact Virus Precipitation Reagent (Invitrogen™, 10720D), resuspended in phosphate-buffered saline (PBS 1X), and kept frozen at −80° C until use. Plaque assay was used to determine the viral titer by incubating serial dilutions of infected supernatant on Vero E6 monolayers at 37 °C and 5% CO_2_ for 1 h. Then the inoculum was removed, cells were washed with PBS 1X and overlaid with DMEM 2% FBS containing 1.5% carboxymethylcellulose sodium salt (CMC, Sigma-Aldrich, St Louis, USA, C5013) for 3 days at 37 °C and 5% CO2. The cells were then fixed with 3.7% paraformaldehyde (PFA, Sigma-Aldrich, St Louis, USA, 158,127) and stained with 1% crystal violet (Sigma-Aldrich, St Louis, USA, C6158).

Cultured iPSC astrocytes and HMC3 cells were mock-infected or infected at of 5 for 2 h at 37 °C and 5% CO2. Primary neuronal rat cultures were mock-infected and infected at MOI 1 with SARS-CoV-2 or exposed to UV-inactivated virus for 2 h at 37 °C and 5% CO2. Then, the inoculum was removed, cells were rinsed once with PBS 1X and fresh medium supplemented with cGAS inhibitors were added to the cultures. cGAS inhibitors were used as follow: human cells were treated with G140 (Invivogen, inh-g140) at a concentration of 25 µM and rat cells were treated with RU.521 (Invivogen, inh-ru521-2) at a concentration of 20 µM.

### Enzyme linked immunosorbent assay (ELISA)

A complete panel of cytokines and chemokines was evaluated on primary neuronal rat cultures using 96 well Rat Cytokine ELISA Plate Array (Signosis, Santa Clara, CA, EA-4006) coated with 16 specific cytokine capture antibodies, following the manufacturer’s instructions. The cytokine profile was obtained from the supernatant of all three culture conditions: mock-infected negative controls, SARS-CoV-2-infected cultures and UV-SARS-CoV-2 control cultures.

### Real-time quantitative reverse transcription PCR (RT-qPCR)

RT-qPCR was performed from total cellular RNA extracted with Total RNA Purification kit according to the manufacturer’s protocol (Norgen Biotek corp, 17,250). Then, 200 ng were reverse-transcribed with SensiFAST cDNA Synthesis kit (Meridian, BIO-65054). Quantification of mRNA was obtained by real-time PCR using the Kapa Sybr fast qPCR kit (Roche, KK4600) on a CFX96 Bio-Rad thermocycler. Data shown are the relative amount of the indicated mRNA derived from human or rat cells normalized to that of glyceraldehyde-3-phosphate dehydrogenase (GAPDH). Quantification of the genomic copies of viral RNA was determined as previously described by (Rajasekharan et al. 2021).Primers are listed in Table 1.

**Table 1:** List of primers

Target	Forward sequence (5’- > 3’)	Reverse sequence (5’- > 3’)	Species
CCL5	GCTGCTTTGCCTACCTCTCC	TTCCTTCGAGTGACAAAGACGA	rat
GAPDH	ATGGTGAAGGTCGGGTGAA	GTTGATGGCAACAATCTCCA	rat
TNF	ATGGGCTCCCTCTCATCAGT	GCTTGGTGGTTTGCTAACGAC	rat
CCL2	AGCCAACTCTCACTGAAGCC	TGGGGCATTAACTGCATCTGG	rat
IFN-β	CAACCTCAGCTACAGGACGG	TCGTGGATGTCACCCAAGTC	rat
DROSTEN N	CACATTGGCACCCGCAATC	GAGGAACGAGAAGAGGCTTG	virus
dsDNA_45	TACAGATCTACTAGTGATCTATGACTGATCTGTACATGATCTACA	TGTAGATCATGTACAGATCAGTCATAGATCACTAGTAGATCTGTA	mouse
ACE2	CCGAAATACGTGGAACTCATCAA	CACGAGTCCCCTGCATCTACA	human
TMPRSS2	CGACAAATGAGGGCAGACGG	ACAAGGGGTTAGGGAGAGCA	human
CCL5	TCATTGCTACTGCCCTCTGC	TACTCCTTGATGTGGGCACG	human
GAPDH	CATGAGAAGTATGACAACAGC	AGTCCTTCCACGATACCAAAG	human
TNF	GCCCATGTTGTAGCAAACCCT	GAGGTACAGGCCCTCTGATG	human
CCL2	CATGAAAGTCTCTGCCGCCC	GGGCATTGATTGCATCTGGCTG	human
IFN-β	AGGACAGGATGAACTTTGAC	TGATAGACATTAGCCAGGAG	human
dsDNA_90	TACAGATCTACTAGTGATCTATGACTGATCTGTACATGATCTACATACAGATCTACTAGTGATCTATGACTGATCTGTACATGATCTACA	TGTAGATCATGTACAGATCAGTCATAGATCACTAGTAGATCTGTATGTAGATCATGTACAGATCAGTCATAGATCACTAGTAGATCTGTA	human

### cGAS stimulation

To activate cGAS pathway, iPSC astrocytes, HMC3 cells and rat cortical cells were transfected with 1 µg of DNA Oligonucleotides (dsDNA) using Lipofectamine LTX (Invitro gen, A12621) following manufacturer’s instructions.

The length of the oligonucleotides used to stimulate human cells was 90 bp (dsDNA 90 bp) and to stimulate rat cells 45 bp (dsDNA 45 bp). After 2 h transfection, HMC3 and iPSC astrocytes were treated with G140 (Invivogen, inh-g140) and rat cortical cultures were treated with RU.521 (Invivogen, inh-ru521-2), as previously described.

### Immunocytochemistry and confocal microscopy

Cultures were fixed in 3.7% PFA solution (Sigma-Aldrich, St Louis, USA, 158,127), permeabilized with 0.1% Triton™ X-100 (Sigma-Aldrich, St Louis, USA, 282,103), and then were blocked for 1 h at 37 °C with PBS 1X supplemented with 1% bovine serum albumin fraction V (BSA, Roche,0.10735078001) and 0.1% TWEEN®20 (Sigma-Aldrich, St Louis, USA, P2287). After blocking, fixed cultures were incubated overnight at 4 °C in a humidified chamber with primary antibodies (Table 2). Then, the cultures were washed three times with PBS 1X supplemented with 0.1% TWEEN®20 (Sigma-Aldrich, St Louis, USA, P2287) and incubated again with the secondary antibodies (Table 2) for 1 h at 37 °C in humidified chamber. Stained cultures were examined using a Zeiss 880 inverted confocal microscope with a 63X oil immersion objective Zeiss 880 Airyscan confocal (Zeiss, Oberkochen, Germany).

**Table 2:** List of antibodies

Name	cat.no	Provider	RRID	Host
glial fibrillary acidic protein (GFAP)	G3893	Sigma-Aldrich	AB_477010	mouse
β-tubulin III (TUJ1)	T3952	Sigma-Aldrich	AB_1841226	mouse
IBA1	019–19741	Wako	AB_839504	rabbit
SARS-CoV-2 Nucleocapsid	40,588-T62	SinoBiologicals	AB_3064900	rabbit
γH2AX (Ser139)	05–636	Millipore	AB_309864	rabbit
P21	#2946	Cell Signaling Technology	AB_2260325	mouse
cGAS	#15,102	Cell Signaling Technology	AB_2732795	rabbit
SARS-CoV-2 Nucleocapsid (1A6)	MA5-35,941	ThermoFisher Scientific	AB_2866553	human
NeuN (E4M5P)	#94,403	Cell Signaling Technology	AB_2904530	mouse
Synapsin-1 (D12G5)	#5297	Cell Signaling Technology	AB_2616578	rabbit
NF-κB p65 (D14E12)	#8242	Cell Signaling Technology	AB_10859369	rabbit
Phospho-NF-κB p65 (Ser536)	#3033	Cell Signaling Technology	AB_331284	rabbit
TBK1/NAK (D1B4)	#3504	Cell Signaling Technology	AB_2255663	rabbit
Phospho-TBK1/NAK (Ser172)	#5483	Cell Signaling Technology	AB_10693472	rabbit
F(ab')2-Goat anti-Mouse IgG (H + L) Cross-Adsorbed Secondary Antibody, Alexa Fluor™ Plus 488	A48286	ThermoFischer Scientific	AB_2896351	goat
Donkey anti-Rabbit IgG (H + L) Highly Cross-Adsorbed Secondary Antibody, Alexa Fluor™ 594	A21207	ThermoFischer Scientific	AB_3697796	donkey
Goat anti-Human IgG (H + L) Cross-Adsorbed Secondary Antibody, Alexa Fluor™ 647	A-21445	ThermoFischer Scientific	AB_3697798	goat

### Immunofluorescence analysis

All image analysis were performed using Fiji tools (Schindelin et al. 2012). In the case of immunofluorescence analysis of cGAS, P21 and γH2AX markers in iPSC astrocytes, microglia cells (HMC3) and rat cortical cultures, percentage of positive and negative cells was calculated in > 200 cells. Total number of cells was determined by counting the number of nuclei, for each experimental condition. To this purpose, we analyzed around 10 to 20 images for each independent experiment (N = 3). Due to the complex morphology of the rat cortical cultures, we measure infection by determining a yellow threshold color, which is the combination within green (GFAP and β-tubulin III signal) and red (Nucleocapsid protein of SARS-CoV-2 signal). Total color threshold was then used to determine the percentage of yellow threshold on each image. More than 20 images were analyzed for each experiment (N = 3). Lastly, Synapsin-1 signal in the rat cortical culture, infected vs non-infected, was determined by using the image calculator for the analysis of particles (puncta) on the entire field of 10 to 20 images per condition. A total of 3 experimental replicates were analyzed and the operator was blind to the experiments for unbiassed counting.

### Immunoblotting

Whole cell extracts were obtained by lysing HMC3 in 1 × RIPA buffer (0.1% SDS, 1% Nonident P-40, 0.5% DOC, 150 mM NaCl, 1 mM EDTA, 1 mM EGTA, 1 mM PMSF, 50 mM Tris–Cl pH 7.5 and Protease and Phosphatase inhibitor). Before fractionation on 10% gradient SDS–PAGE, whole extracts were boiled and sonicated for 10 min at low intensity using Ultrasonic Cleaner (3510E-MT;Branson) in a water bath at 4 °C. Proteins were then transferred onto nitrocellulose membrane and probed over-night with primary antibodies listed in Supplementary Table 1. Blot imaging was performed with a chemiluminescence imaging system (Alliance-LD2-87.WL; Uvitec) and quantification of protein levels was conducted by densitometric analysis with ImageJ 1.53a.

### MEA recordings and electrical stimulation

MEA recordings on infected neurons were performed within a BSL-3 laboratory. We used commercial MEAs (Multichannel Systems GmBH, Reutlingen, Germany) to monitor the extracellular electrical activity of the primary neuronal cultures. Each MEA contains 120 titanium nitrate (TiN) microelectrodes with diameters of 30 μm and an inter-electrode distance of 100 μm. Each microelectrode detected extracellular action potentials from neurons located in their proximity. We employed an electronic multichannel amplifier (MEA2100-Mini-120-System, Multi-Channel Systems) with 10–10000 Hz bandwidth and an amplification factor of 1. Recordings were performed inside a (dry) incubator, at 37 °C and 5% CO2. Evaporation and osmolarity drifts were strongly reduced by sealing MEAs with disposable PDMS caps (Blau, et al., 2009). Extracellular raw electrical signals were then sampled at 25 kHz/channel and digitized with a 16-bit resolution by the MEA2100-Mini USB interface. The raw voltage traces were analyzed by custom scripts written in Julia, an updated version of a MATLAB-based framework for parallel preprocessing previously reported (Mahmud et al. 2014). These were used to extract the time of occurrence of action potentials at each MEA microelectrode and perform a series of analysis. Briefly, for each recording channel, a threshold for peak detection was set depending on the background electrical noise (Quiroga et al. 2004). Each recording channel was considered as corresponding to an active electrode if the detected spiking activity at that electrode exceeded the rate of 0.02 Hz. We then identified the occurrence of network-wide bursts of action potential, the collective spiking events occurring synchronously across at least 10% of the active electrodes and within a 25 ms time window, each detected with a dead time interval of 100 ms. For the extracellular electrical stimulation of neuronal activity, we applied 10 voltage-driven biphasic square pulses of 600 μV peak-to-peak amplitude, delivered every 6 s through 2 active microelectrodes (i.e., bipolar configuration). We recorded the extracellular electrical activity for 1 min during and after each stimulus.

### Recombinant RBD protein production

To produce a recombinant RBD-Spike protein in vitro, the protocol published by Daniel Stadlbauer and colleagues in 2020 (Stadlbauer et al. 2020) was applied and adapted. Briefly, 35 × 10^6^ HEK293T/17 cells (ATCC CRL-11268) were plated in ten 150 mm dishes, kept in culture and transfected when ~ 85% confluent. For transfection, linear polyethilimine (PEI, Sigma-Aldrich, 764,604) and a plasmid with pCAGGS backbone containing the SARS-CoV-2, Wuhan-Hu-1 spike glycoprotein gene RBD with C-terminal hexa-histidine tag were used keeping a ratio of 1 µg of DNA: 11 µl of PEI. The day after fresh media was replaced and the protocol for protein purification was conducted three days post transfection. Briefly, the Spike protein was isolated through metal-chelate affinity chromatography. Cells media (200 mL) was filtered through a 0.22 µm filter (Millipore, S2CGPU05RE) and incubated with Ni–NTA resin (Qiagen, 30,210) for two hours at room temperature while shaking (65 rpm). Then, two clean 5-ml polypropylene columns were loaded with the filtered media-resin mixture and then washed with one column volume of wash buffer four times. Four fractions were eluted from each column by incubating the resin in the column with 3 ml of elution buffer for each fraction. After that, the eluate was put through 50-kDa Amicon Ultra Centrifugal Filter Units at 4000 × g for 30 min until only 200 to 300 μl remain in the unit. Finally, the protein concentration was measured, and a denaturing SDS-PAGE was run to check the integrity of the purified protein.

### Data analysis

All the data obtained across the experiments were analyzed and reported graphically with GraphPad (Insight Venture Management, New York, US). We performed 3–5 biological replicates, each with at least two technical replicates in order to be in the range of an acceptable 10–20 error degree of freedom according to the Resource Equation Method to determine the appropriate sample size. Randomization methods were not needed for in vitro experiments. Then, we tested the normality of the data set by using Shapiro–Wilk or Anderson–Darling test followed by Levene’s test to determine homogeneity of the variance (supplementary Table 1). Normally distributed datasets were tested either with one-way ANOVA followed by Dunnett’s, Tukey’s, Bonferroni’s or Holm-Sidak corrected multiple comparison test or with T-test for single comparisons. For nonparametric samples, Kruskal–Wallis, Mann–Whitney or Friedman test were used for analysis. Bursts and spike-trains analysis was performed with custom MATLAB scripts, focusing on the cumulative distributions of three parameters: the average number of bursts, the Inter-Burst Intervals (IBIs), and the Burst durations (BDs).

## Results

### SARS-CoV-2 infection of human astrocytes leads to DNA damage and induction of the cGAS pathway

Astrocytes are the first line of defense against viruses in the brain. Human induced pluripotent stem cells (iPSC) were used to produce astrocytes, as already reported in Padmashri et al. (Padmashri et al. 2021). Infection with ancestral SARS-CoV-2 was performed at a multiplicity of infection (MOI) of 5 and cells were treated with the specific cGAS inhibitor (G140) after 2 h post infection (hpi). At 48 hours (h) we confirmed infection and checked for markers of: senescence, virus induced DNA damage (VID), downstream activation of the cGAS pathway and expression of relevant antiviral chemokines and cytokines.

**Fig. 1:**
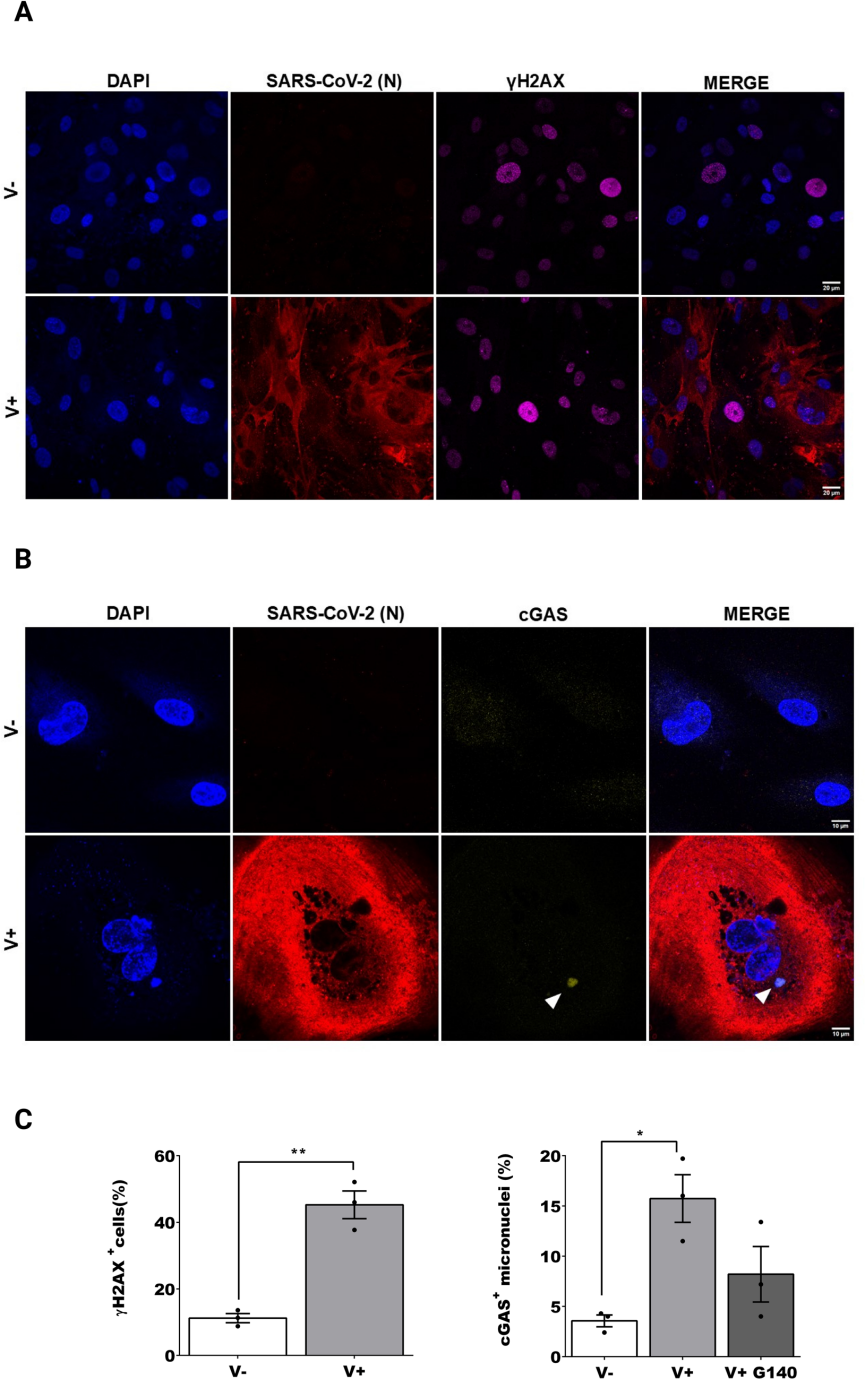
**SARS-CoV-2 infection of iPSC astrocytes induces a DNA damage response and cGAS micronuclei**. Immunofluorescence (IF) images of non-infected (V −) or infected (V +). **A** IF of V- or V + iPSC astrocytes positive to γH2AX (N in red, γH2AX in magenta), unpaired sample T-test. **B** IF of V-, V + iPSC astrocytes positive to cGAS micronuclei (N in red, cGAS in yellow), one-way ANOVA followed by Holm-Sidak method in case of multiple comparisons**. C** Quantification of the percentage of cells positive to γH2AX or cGAS micronuclei, n > 200. All samples were fixed at 48 hpi and nuclei were stained with DAPI. Scale bar, 10 μm or 20 μm. For each figure the average values are shown with standard error of means (SEM) and p-values. All experiments were performed in triplicates and only significant P-values are indicated by the asterisks above the graphs (*P < 0.05, **P < 0.01)

We show (Fig. 1 A and B) that iPSC astrocytic cultures are susceptible to SARS-CoV-2 as demonstrated by immunofluorescence (IF) of Nucleocapsid protein of SARS-CoV-2 (N) and by western blot (WB) (supplementary Fig. 1 A). Replicative virus particles were measured by plaque assay (plaque forming units per milliliter, PFU/mL). After 48 h viral load in supernatant of infected cells was 2.3 × 10^4^ PFU/mL. We also investigated infection of HMC3 at the same MOI as for iPSC astrocytes. At 24 h, we observed a few positive cells for N protein (supplementary Fig. 1 D and E) and a low level of infectious virus in the supernatant of infected cells detected by plaque assay (5 × 10^2^ PFU/mL).Furthermore, we quantified viral genomes (N gene RNA copies/µL) from cell lysates of infected iPSC astrocytes and HMC3 at time 0 and at 48 and 24hpi, respectively (supplementary Fig. 1 B). We observed a significant increment of 2 log in iPSC astrocytes at 48 hpi *(one-way ANOVA followed by Dunnett’s method, F (2, 6)* = *6.832, P* = *0.0284, V* + *0 h vs. V* + *48 h P* = *0.0272, V* + *0 h vs. V* + *G140 48 h P* = *0.0405).* No differences were observed in HMC3 lysates, indicating a non-productive infection (supplementary Fig. 1 B). Then, we analysed the expression of *ACE2* and *TMPRSS2* in both cell types (supplementary Fig. 1 C). The expression of *ACE2 * increased in iPSC infected astrocytes compared to non-infected cells *(one-way ANOVA followed by Dunnett’s method, F (2, 6)* = *7.638, P* = *0.0224, V- vs. V* + *P* = *0.0280, V- vs V* + *G140 P* = *0.0244)*. Moreover, there is a significant difference when comparing the basal expression of *ACE2* in iPSC astrocytes and HMC3 *(two-way ANOVA followed by Sidak’s test, F (2, 4)* = *29,33, P* = *0.0041, V* + *P* = *0,0135, V* + *G140 P* = *0.0054).* No significant difference could be found for the levels of *TMPRSS2*, which showed a pattern similar to *ACE2* (supplementary Fig. 1 C). supplementary Fig. 1 C).

DNA damage response (DDR) is a common factor that may lead to cellular senescence. To this end, we investigated the formation of γH2AX foci in the nucleus, which is an early marker for double strand DNA breaks and repair. We observed a significant increase *(T-test, t(4)* = *7.740, P* = *0.0015)* of γH2AX foci in infected iPSC astrocytes compared to mock infected controls (Fig. 1 A and C). Genomic stress leads to the accumulation of activated cGAS/DNA in cytoplasmic micronuclei. While only 15.7% of infected cells presented cGAS micronuclei, these were reduced to 8.2% when cells were treated with the G140 inhibitor (Fig. 1 C) *(one-way ANOVA, F (2, 6)* = *8.329, P* = *0.0186, adjusted P Value: V- vs. V* + *P* = *0.0202)*. In line with the previous observation, we detected an increment *(T-test, t(4)* = *6.347, P* = *0.0032)* of γH2AX foci in infected HMC3 (supplementary Fig. 1 D) but not in the number of micronuclei positive for cGAS (supplementary Fig. 1 E).

### SARS-CoV-2 infection of human astrocytes induces expression of *IFN-β* and cellular senescence

**Fig. 2:**
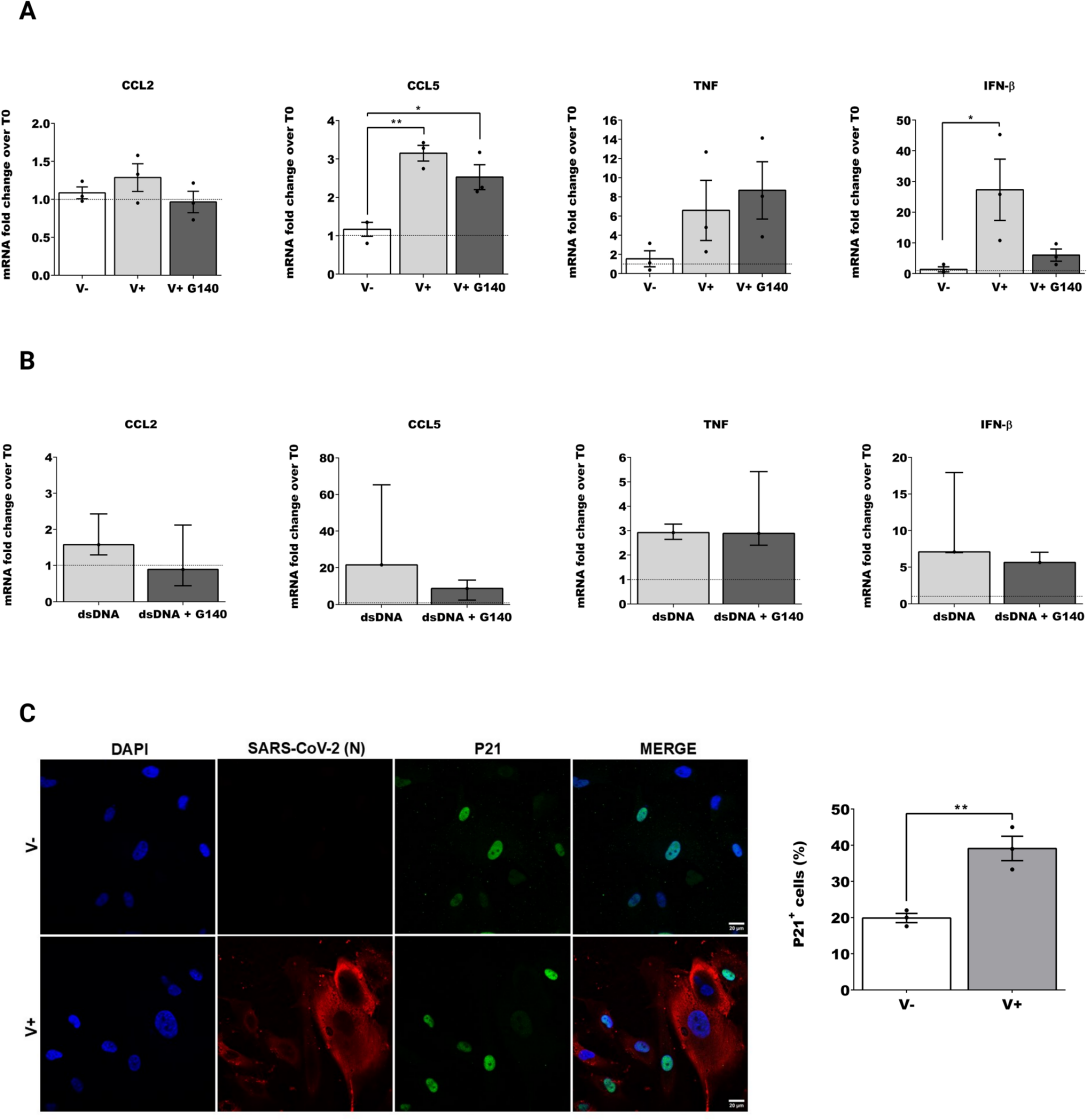
***SARS-CoV-2 infection of iPSC astrocytes induces pro-inflammatory pathways. A*** Quantitative RT–qPCR was used to quantify the fold change in the expression levels of RNA comparing time zero with 48 hpi for: chemokine C–C motif ligand 2 (*CCL2*) and chemokine C–C motif ligand 5 (*CCL5*), tumor necrosis factor α (*TNF*), interferon β (*IFN-β*). In white V-, in light grey V +, in dark grey V + G140. One-way ANOVA followed by Tukey’s method in case of multiple comparisons. **B** Quantitative RT–qPCR was used to quantify the fold change in the expression levels of RNA comparing time zero to 48 hpi for *CCL2*, *CCL5*, *TNF* and *IFN-β*. In light grey dsDNA and in dark grey dsDNA + G140. Non-parametric Mann Whitney reported as mean and interquartile range. **C** Immunofluorescence (IF) images and quantification of non-infected (V −) or infected (V +) iPSC astrocytes positive to P21 (SARS-CoV-2 nucleocapsid (N) in red, P21 in green), unpaired parametric T-test. For each figure the average values are shown with SEM, all experiments were performed in triplicates and only significant P-values are indicated by the asterisks above the graphs (*P < 0.05, **P < 0.01)

Viral infection ignites complex mechanisms of activation, coordination, and regulation of the immune response where cytokines and chemokines play crucial roles. The cGAS-STING pathway activates antiviral immune responses by promoting the expression of interferons (IFNs) as well as virus-induced cytokines and chemokines. We measured the relative expressions of *CCL2*, *CCL5*, *TNF* and *IFN-β* 48 hpi. In iPSC astrocytes, *IFN-β* dramatically increased after infection with SARS-CoV-2 *(one-way ANOVA followed by Tukey’s method F (2, 6)* = *5.495, P* = *0.0440, V- vs. V* + *, P* = *0.0475)* and was blocked by the cGAS inhibitor G140 indicating a possible contribution of this pathway to the antiviral response (Fig. 2 A). IFN-β is known to contribute to the protective response to viral infection in the CNS by in turn regulating IFN-γ-dependent responses (Hwang and Bergmann 2018), and its release contributes to astrocyte activation itself (Clarke et al. 2019), suggesting the beginning of a cascade of astrocytes activating in response to the infection. The increase in *IFN-β* expression could indicate an activation of the astrocytes that release *IFN-β* to further activate glial cells, compatibly with findings in literature that astrocytes releasing IFN-β is a classic pathway of viral infection response in the CNS (Hwang and Bergmann 2018; Clarke et al. 2019).

Although the expression of *CCL2, CCL5* and *TNF* was not as robust as *IFN-β*, there was a pattern of activation after infection, which was mildly inhibited by G140 for *CCL2* and *CCL5* (Fig. 2 A). In the case of *CCL5,* we observed statistically significant differences within non infected and infected cells *(one-way ANOVA followed by Tukey’s method, F (2, 6)* = *17.10, P* = *0.0033, V- vs. V* + *P* = *0.0030, V- vs. V* + *G140 P* = *0.0184)*. Astrocytes are significant sources of classical pro-inflammatory chemokines such as CCL2 and CCL5 (Croitoru-Lamoury et al. 2003). Overexpression of *CCL2* can promote the increase of harmful glial neuroinflammatory changes (He et al. 2016; Joly-Amado et al. 2020). On the other hand, CCL5 produced by murine astrocytes induces the expression of multiple cytokines and chemokines (Luo et al. 2002) which in turn may contribute to the neuroinflammatory environment after SARS-CoV-2 infection. Minor changes on *TNF* expression were observed at 48 h after infection (Fig. 2 A). TNF is a well know key player during viral infection by regulating inflammatory responses (Domm et al. 2008), as well as chemoattractant of neutrophils (Vieira et al. 2009). However, aberrant production could lead to severe inflammatory cytokine storms. In HMC3, we observed a significant increment of the expression of cytokines *CCL2 (one-way ANOVA followed by Holm-Sidak F (2, 6)* = *7.334, P* = *0.0245, V* + *vs. V* + *G140 P* = *0.0284)* and *CCL5* (*one-way ANOVA followed by Holm-Sidak, F (2, 6)* = *6.709, P* = *0.0295, V- vs. V* + *P* = *0.0339)* after infection, with a reduced expression following G140 treatment (supplementary Fig. 2 A).

To validate our observations, we transfected both cell types with dsDNA 90 bp (Xia et al. 2019), which is a direct agonist of cGAS. In iPSC astrocytes, we observed an increment of *IFN-β, CCL2* and *CCL5* expression after dsDNA transfection, which was inhibited by G140 (Fig. 2 B). Again, in HMC3 we observed an induction of the expression of the cytokines and chemokines after dsDNA 90 bp transfection (supplementary Fig. 2 B).

We studied the contribution of the key kinases TANK-binding kinase 1 (TBK1) and nuclear factor κB (NF-κB) (Supplementary Fig. 3). TBK1 is an enzyme that phosphorylates STING, leading to recruitment of interferon regulatory factor-3 (IRF3), which translocate into the nucleus to induce transcription of ISGs, cytokines, interferons, and chemokines genes (Shu et al. 2014; Fang et al. 2017). Moreover, TBK1 and its homolog IκB kinase epsilon (IKKε) lead to activation of the IKK complex, which then activates the transcription factor NF-κB (Balka et al. 2020). NF-κB synergizes with IRF3 to induce high levels of type I IFN and other pro-inflammatory cytokines (Smale 2010). Therefore, cell lysates of infected iPSC astrocytes and HMC3 cells were collected for the detection of total and phosphorylated NF-κB and TBK1. As a positive control of the phosphorylation of NF-κB and TBK1 we used poly I:C, which is a synthetic molecule that induce an innate immunity response via MDA5 receptor (Kato et al. 2008). After infection, both cell types showed an increment of the total protein levels of NF-κB and TBK1 in infected cells (supplementary Fig. 3). However, the phosphorylated form of both proteins was marginal in both cell types with a slight increment in microglia infected cells. Moreover, the increment of total TBK1 and NF-κB protein was partially reduced after addition of G140 in both cell types (supplementary Fig. 3). Since the convergence of RNA and DNA sensing pathways may contribute to the antiviral response (Zevini et al. 2017), additional data would be required to further dissect the molecular pathways of inflammatory signaling.

An increment in pro-inflammatory responses is indicative of persistent senescence. Therefore, we analyzed the specific marker of senescence P21 by immunofluorescence showing a significant increment *(T-test, t(4)* = *5.329, P* = *0.0060)* in virus infected iPSC astrocytes (Fig. 2 C). Accordingly, HMC3 microglia showed a higher percentage of P21 *(T-test, t(4)* = *5.218, p* = *0.0064)* positive infected cells compared to mock (supplementary Fig. 2 C.

The results obtained with SARS-CoV-2 infection of astrocytes point to a pro-inflammatory antiviral immune response triggered by the activation of the cGAS-STING pathway following virus induced DNA damage, as observed by the increment of γH2AX positive cells, and higher percentage of senescence cells. Moreover, activated microglia produces inflammatory cytokines, boosting the inflammatory tissue damage. Taking together these results we can hypothesize that human glial cells are key to initiate the inflammatory cascade in the brain.

### SARS-CoV-2 infection of rat cortical cells ex vivo targets principally astrocytes

To study the cellular response to infection in a more physiological model we infected dissociated primary cultures of rat neocortex ex vivo and analyzed cells and supernatant at 3, 6 and 24 hpi. To the best of our knowledge, this is the first study that shows that SARS-CoV-2 could infect rat cells.

**Fig. 3:**
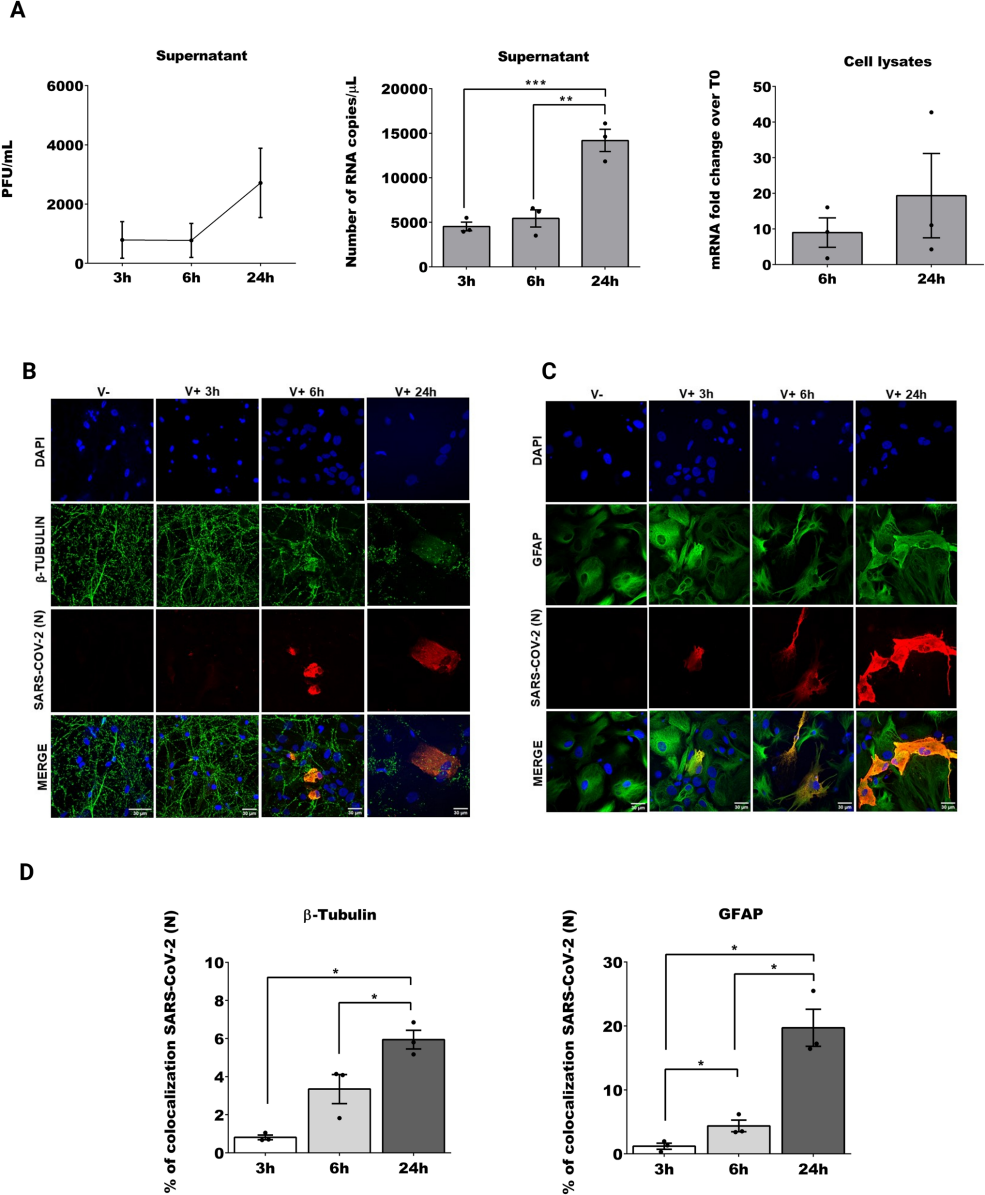
***SARS-CoV-2 infection in primary rat cortical cultures causes synaptic decrease and neuronal loss***. **A** Viral yields measured by plaque assay (PFU/mL), paired non parametric Friedman reported as mean and interquartile range, and number of copies of SARS-CoV-2 RNA (N) per microliter of supernatant, at different hpi, one-way ANOVA followed by Tukey’s method in case of multiple comparisons, and mRNA fold change over T0 in cell lysates, parametric paired T-test. **B** Representative images of primary cortical cultures V- and V + at different hpi. First row shows DAPI in blue, second row shows β-tubulin III in green, third row shows the SARS-CoV-2 (N) in red. **C** V- and V + at different time points, second row shows GFAP in green third row shows the SARS-CoV-2 (N) in red. **D** Quantification of nucleocapsid of SARS-CoV-2 (red) together with β-tubulin III (green) or GFAP (green), parametric paired T-test. For each figure the average values are shown with SEM, all experiments were performed in triplicates and only significant P-values are indicated by the asterisks above the graphs (*P < 0.05, **P < 0.01)

Viral replication kinetics were examined by plaque assay and reported as titers of PFU/mL at different hpi (Fig. 3 A). We also measure the presence of the virus by RT-qPCR in supernatant and cell lysates, and we observed increased viral production *(one-way ANOVA followed by Tukey’s method, F (2, 6)* = *31.17, P* = *0.0007)* from 3 to 24 hpi *(adjusted P Value, P* = *0.0009)* and from 6 to 24 hpi *(adjusted P Value, P* = *0.0016),* demonstrating the ability of SARS-CoV-2 to infect and replicate in rat neocortex (Fig. 3 A). The increment between 6 and 24hpi in cell lysates was non-significant.

To study the tropism of SARS-CoV-2 infection for the different cell types present in a primary rat cortical culture, we analyzed the infection efficiency in neurons and glial cells separately (Fig. 3 B and 3 C, respectively). We classified the efficiency of infection by co-staining SARS-CoV-2 nucleocapsid (N) with GFAP or β-tubulin III (Fig. 3D). Markers of infection were present as early as 3 hpi to increase over time until 24 hpi both in glial cells *(T-test, 3 h vs 6 h: t(2)* = *5.447, P* = *0.0321, 3 h vs 24 h: t(2)* = *7.398, P* = *0.0178, 6 h vs 24 h: t(2)* = *7.718, P* = *0.0164)* and neurons *(T-test, 3hpi vs 24hpi: t(2)* = *8.628, P* = *0.0132, 6 h vs 24 h: t(2)* = *5.468, P* = *0.0319)*. Interestingly, we observed a higher infection rate in GFAP than β-tubulin positive cells (Fig. 3D). Hence, virus replication not only increases over time but also occurs to a higher degree in glial cells, which is in line with the results described above where human iPSC astrocytes showed a high susceptibility to SARS-CoV-2 infection.

We observed a clear decrease of β-tubulin III marker after infection prompting us to check the functionality of the synapsis of the neurons. To this end, we measured in two experimental replicates Synapsin 1, which is a phosphoprotein that participates in maintaining the stability and organization of synaptic vesicles (De Camilli et al. 1983), and NeuN, a protein localized in nuclei and perinuclear cytoplasm of most of the neurons used to identify pathological changes in neuronal populations (Gusel’nikova and Korzhevskiy 2015). In both cases we observed a general decrease of the signal at 24 hpi (supplementary Fig. 4 A). We measured the number of Synapsin 1 puncta, mean size of the puncta, and intensity of the signal. There was an increase of the events in the non-infected cells, but the opposite was observed in the infected cells. Moreover, there was a higher mean of the average size of the puncta in non-infected cells and higher intensity of the signal compared to infected cells (supplementary Fig. 4 A). As well the signal of NeuN marker decreased over time on infected cells, which is in line with the reduced Synapsin 1 signaling, meaning that there is a general loss of neurons along SARS-CoV-2 infection (supplementary Fig. 4 A). However, non-statistically significant results were observed.

### SARS-CoV-2 first decreases synaptic connections and then causes neuronal loss

Once we established the model of infection in rat cortical cultures, we then investigated the effect of SARS-CoV-2 on the electrical activity of neurons by growing neuronal cortical cultures on multi-electrode arrays (MEAs) (Gerber et al. 2021). The electrical activity of the neurons can be recorded by the microelectrodes directly in contact with the cells kept sealed and sterile at 37 °C and 5% CO_2_. We plated 3 cultures on a total of n = 6 MEAs and cultured them in vitro for 21 days. At this stage, in vitro neurons are known to re-grow functional synaptic connections and to develop a mature pattern of spontaneous electrical activity (Kobayashi et al. 1993). A well-known phenomenon of in vitro networks is the irregular occurrence of synchronized, global firing events across the network, referred to as network bursts (from now on, “bursts”). This phenomenon arises from the interplay between recurrent excitatory synaptic activity within the network and intrinsic synaptic fatigue (Giugliano et al. 2004) and is mediated by different receptors among which N-methyl D-aspartate (NMDA) and Gamma-aminobutyric acid (GABA) (Bonifazi et al. 2009; Pozzi et al. 2020).

To test the effect of the virus on neurophysiology, rat cortical cells were seeded on MEAs and later infected at MOI 1 with the ancestral SARS-CoV-2 variant (V +). At the same time, n = 3 control MEAs were exposed to the same amount of SARS-CoV-2 virus that was previously inactivated by exposing it to UV light for 30 min (UV).

**Fig. 4:**
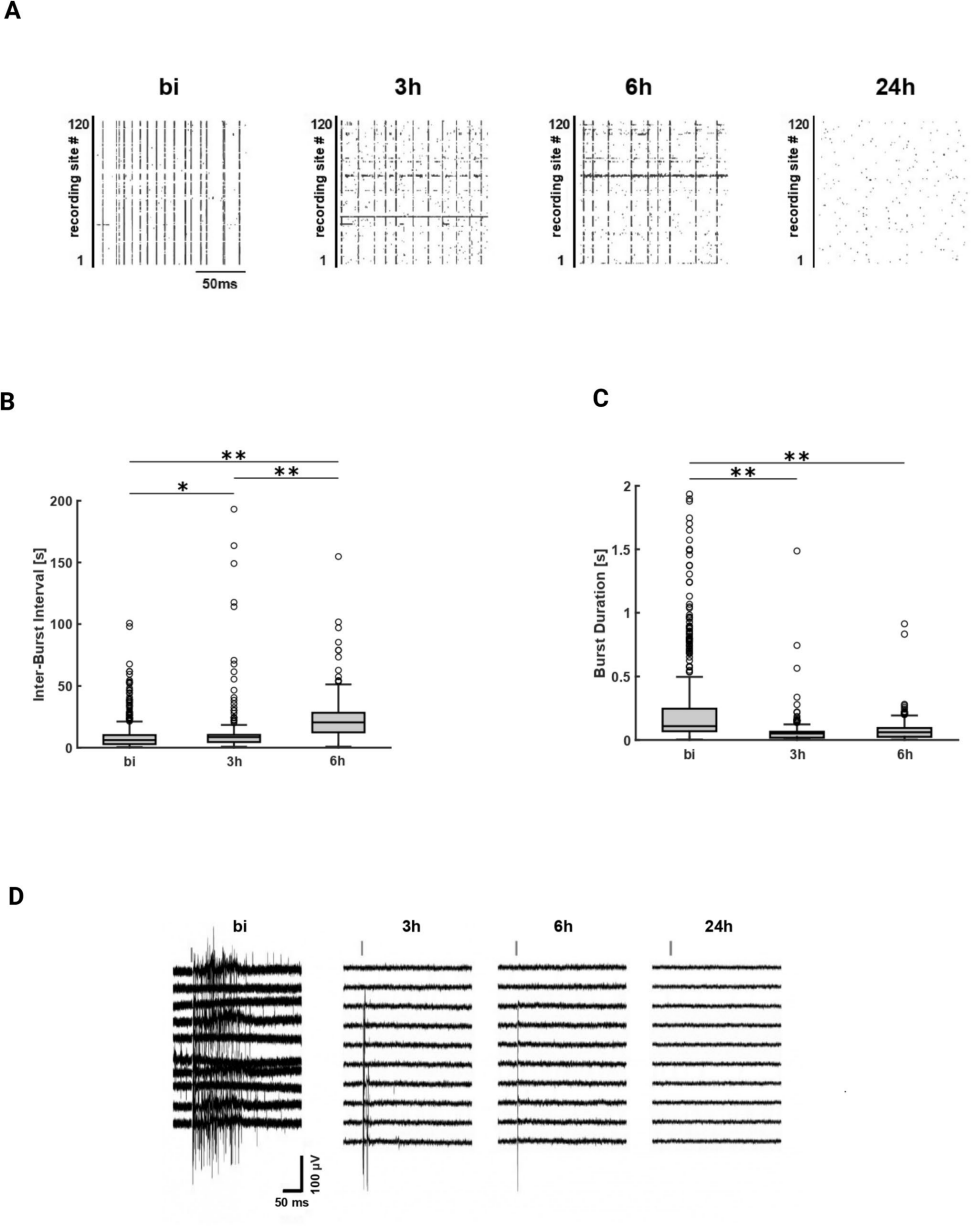
***Loss of electric activity in rat cortical cells after SARS-CoV-2 infection. A*** Representative raster plots of spike times across 120 recording sites (corresponding to the microelectrodes) during 150 ms time windows for four timepoints: before infection (bi), then 3, 6 and 24 h post-infection (h). **B** Inter-burst intervals (IBIs) computed in the same 30-min time windows bi, 3 h and 6 h; The IBIs increased significantly over time (*one-way ANOVA, F(2, 1178)* = *37.22, p* = *2.11* × *10*^*–16*^*)*; in particular, Bonferroni-corrected post-hoc comparison showed that IBIs at 6 h were lower than at 3 h *(P* = *5.87* × *10*^*–9*^*)* and bi *(P* = *9.18* × *10*^*–17*^*)* and IBIs at 3 h were lower than bi *(P* = *4.4* × *10*^*–3*^*)*. **C** Burst Duration (BD) in the same 30-min time window bi, 3 h and 6 h; BD significantly decrease over time *(one-way ANOVA, F(2, 1188)* = *20.10, p* = *2.61* × *10*^*–9*^*)*; in particular, Bonferroni-corrected post-hoc comparison showed that bi was longer than 3 h *(P* = *1.78* × *10*^*–8*^*)* and 6 h *(P* = *3.11* × *10*^*–4*^*)* while 3 h and 6 h were not significantly different *(P* = *1)*. **D** Representative raw voltage traces of neuronal signals after electrical stimulation (600 mV voltage pulses for 100 μs) from ten electrodes at four timepoints: bi, 3, 6, 24 h; the grey vertical bar represents the moment the stimulus is delivered

For each experiment, two cultures, one SARS-CoV-2 and one UV-inactivated SARS-CoV-2 were recorded at the same time before infection and then at regular intervals for the following 24 h. We observed a progressive decrease in the occurrence of both single action potentials (conventionally referred to as “spikes”) and bursts, as shown in the representative raster plot in Fig. 4 A. In the raster plot, each dot represents a spike, happening at a specific time point. The vertical alignment of several dots means that a spike was detected by many electrodes at the same time across the entire surface covered by the electrodes and is thus classified as a burst.

In SARS-CoV-2 cultures, we could still observe a few spontaneous spikes and bursts up to 6 hpi, but not at later timepoints. We did not observe activity at 24 hpi. Two parameters were analyzed: the burst frequency (measured as inter-burst interval, IBI) and the burst duration (BD). The distribution of IBIs and BDs was analyzed before the infection (bi), at a time window around the moment the electrical activity could not be detected anymore (6hpi) and at an intermediate timepoint between the two (3hpi). Figure 4B shows an increase in IBIs duration over time (with median ± IQR values of 6.14 ± 11.08 s bi, 8.56 ± 29.36 s 3 h, and 20.48 ± 20.88 s 6 h) meaning that the occurrence of bursts became less frequent. We found a significant difference among the distributions in the time windows considered *(one-way ANOVA, F(2, 1178)* = *37.22, P* = *2.11* × *10*^*–16*^*)*. The analysis of BDs (Fig. 4C) gave comparable results, i.e. we found a decrease in BD over time (with median values of 0.11 ± 2.34 s bi, 0.05 ± 0.09 s 3 h, and 0.06 ± 0.11 s 6 h). Again, the means of each timepoint were found to be significantly different from the other timepoints *(one-way ANOVA, F(2, 1188)* = *20.10, P* = *2.61* × *10*^*–9*^*)*, implying an impairment of the overall network electrical activity.

The same experimental paradigm was repeated with UV-SARS-CoV-2 control cultures (supplementary Fig. 4B). First, activity was still detectable at 24 h. IBIs showed a trend towards increase between the first two timepoints (*with median values of 7.762* ± *30.556 s bi and 19.257* ± *14.428 s 3 h)* which was then apparently reversed at 6 h (*with a median value of 11.653* ± *18.703 s)*. No significant differences were observed *(one-way ANOVA, F(2, 717)* = *2.48, p* = *8.4* × *10*^*–2*^*)*. BDs remained stable throughout the timepoints *(with median values of 0.097* ± *26.33 s bi, 0.087* ± *0.017 s 3 h, and 0.099* ± *27.689 s 6 h)* and, also in this case, we did not observe any significant difference *(one-way ANOVA, F(2, 563)* = *2.51, p* = *8.3* × *10*^*–2*^*).*

It has been found that purified Spike protein alone can activate inflammatory response in *ACE2* mice (Albornoz et al. 2023). To investigate the ability of the Spike protein to activate inflammation in rat cortical cells, recombinant Spike protein was produced and used to treat cells at 100 nM. Then, a series of recordings on control cultures were performed with the same previously described experimental parameters. In each of the n = 4 MEAs, no significant difference was found in the number of bursts *(Kruskal–Wallis test, H(3)* = *0.5026, p* = *9.2* × *10*^*–1*^*)* between any of the timepoints, and indeed the value remained stable before incubation (1082.5 ± 386.98), 3 h (1185 ± 327.46), 6 h (1035.5 ± 318.63) and activity was always detectable at 24 h (1135.5 ± 413.63 mean number of bursts)(supplementary Fig. 4 C). After recording the spontaneous activity in SARS-CoV-2 cultures, we applied voltage pulses of 600 mV for 100 μs to a small number of active electrodes in the arrays to verify whether synaptic connections were still intact. This type of stimulation should first evoke a “direct” response, which is independent of glutamatergic synaptic activity and should occur within the first 10–20 ms. Evoked responses are most likely the result of antidromic excitation through an axon near the stimulation electrode. Stimulated neurons with an intact glutamatergic synaptic activity will present also a second, longer synaptic response lasting up to 50 ms post-stimulus (Wagenaar et al. 2004). While both responses were intact at the network level before the infection, they decreased in duration after 3–6 h and totally disappeared when the silencing of the overall spontaneous activity was observed (Fig. 4D). These results suggest that, after SARS-CoV-2 infection, first synaptic connection between neurons was progressively lost and only then neuronal death occurred.

### SARS-CoV-2 induce cGAS-STING pathway activation in rat cortical cultures mediating the release of pro-inflammatory cytokines and chemokines

**Fig. 5:**
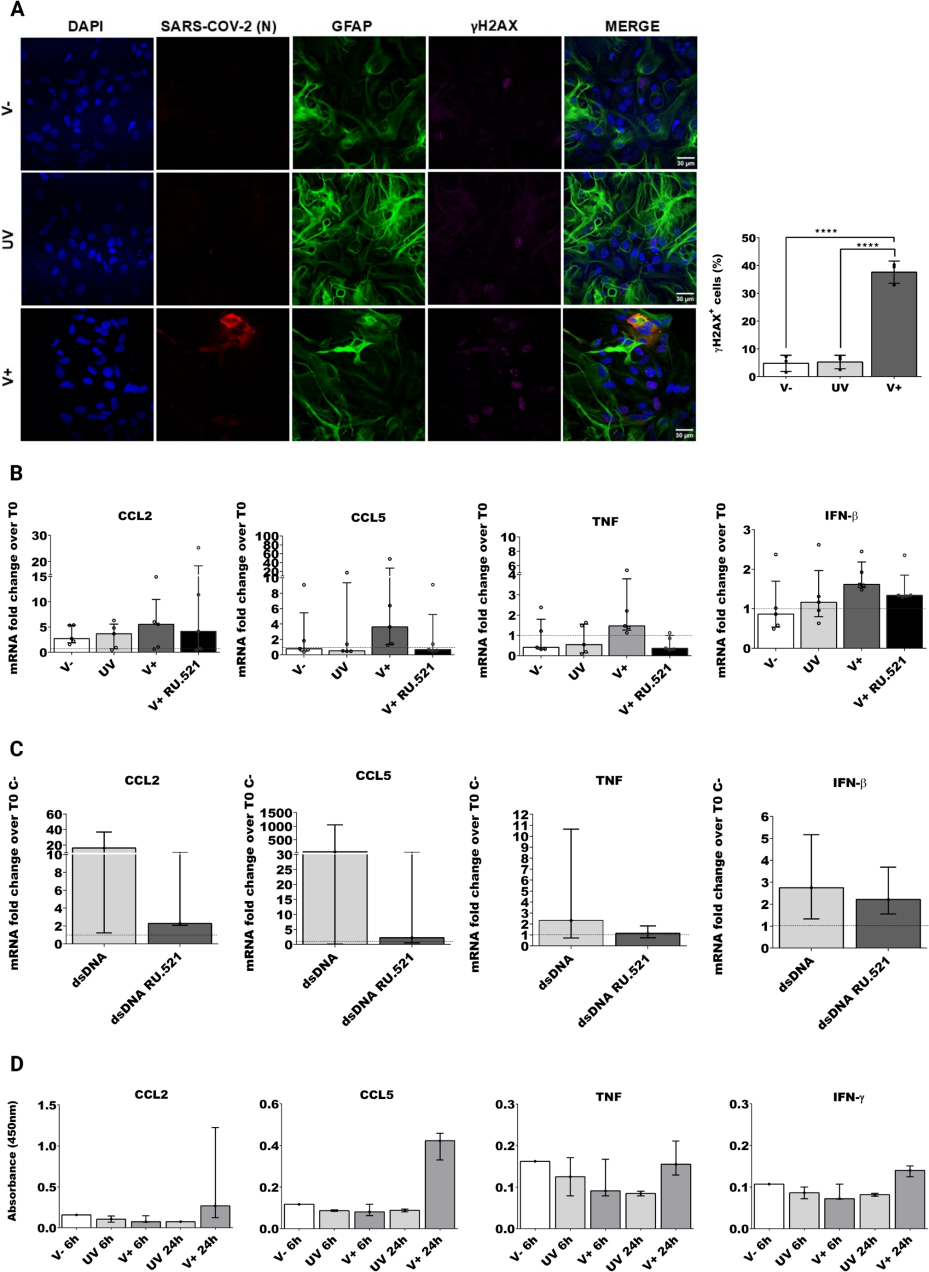
***DNA damage and cGAS involvement in the inflammatory response of infected rat cortical cells. A*** IF of V-, UV, V + rat cortical cultures positive to γH2AX (GFAP in green, N in red, γH2AX in magenta) and quantification of percentage of cells positive to γH2AX in the total cell type population, n > 200, unpaired one- ANOVA and average values are shown with SEM. All samples were fixed at 24 hpi and nuclei were stained with DAPI. Scale bar, 30 μm. **B** Quantitative RT–qPCR was used to quantify the expression levels of RNA for *CCL2*, *CCL5, IFN-β* and *TNF*. The quantification is expressed as the fold change in mRNA expression compared to T0. White is for V-, light grey for UV-SARS-CoV-2 (UV), dark grey for V + and black for V + RU.521, unpaired non-parametric Kruskal–Wallis, reported as mean and interquartile range. **C** Quantitative RT–qPCR was used to quantify the fold change in the expression levels of RNA comparing T0 to 24 h for *CCL2*, *CCL5*, *TNF* and *IFN-β*. In white, dsDNA in light grey (dsDNA + G140), unpaired non-parametric Mann–Whitney, reported as median and interquartile range. **D** Qualitative detection of rat-specific cytokines and chemokines by ELISA. The panel of cytokines and chemokines from the supernatant of V-, UV and V + at 6- and 24-hpi, unpaired non-parametric Kruskal–Wallis. Results are shown by reported median and interquartile range of the absorbance values measured at 450 nm, for V-, n = 1, UV, n = 2 and V + n = 3, unpaired non-parametric Kruskal–Wallis test, reported as median and interquartile range. All experiments were performed in triplicates and only significant P-values are indicated by the asterisks above the graphs (*P < 0.05, ****P < 0.0001)

Following our observations in human astrocytes, we tested the presence of damaged DNA foci in the infected rat cortical cultures. Infection was performed as previously described and the presence of γH2AX foci was determined in three cell types by using β-tubulin III for positive neurons, ionized calcium-binding adapter molecule 1 (IBA1) for positive microglia and GFAP for positive astrocytes (supplementary Fig. 4 D). We studied the presence of γH2AX foci in the total population under the following conditions: non-infected (V-), SARS-CoV-2 infected (V +) and cells cultures exposed to UV-inactivated virus (UV). We analyzed more than 200 cells per condition in 3 biological replicates, independently on the cell type, and we found a higher number of γH2AX-positive cells in SARS-CoV-2 infected cultures *(one-way ANOVA F (2, 6)* = *104.9, P* < *0.0001)* than both non-infected *(adjusted P-value, P* < *0.0001*) and UV cultures *(adjusted P-value, P* < *0.0001)* (Fig. 5 A). Our observation confirms the data from human glial cells that identify SARS-CoV-2 as an inducer of DNA damage response. Mislocated DNA can activate cGAS-STING pathway. Since we could not detect rat cGAS by immunostaining, we decided to study the functional relevance of the pathway in the virus-induced inflammatory response. We infected rat cortical cultures as previously described, treating or not with the specific cGAS inhibitor, RU.521. As reported previously, RU.521 is a potent inhibitor of the mouse cGAS (Vincent et al. 2017), but is a poor inhibitor of human cGAS (Lama et al. 2019). Since RU.521 and G140 are not specific for inhibiting rat enzyme activity, we tested both in the rat cortical cultures and we observed a stronger inhibition with RU.521 (data not shown); therefore, we continued the studies with this drug (Vincent et al. 2017; Lama et al. 2019). Next, we measured the relative expression of viral induced cytokines and chemokines at 24hpi, in five biological replicates of non-infected (V-), infected (V +), cells exposed to UV inactivated virus (UV) and infected cells treated with RU.521 (V + RU.521). In contrast to the observation in human iPSC astrocytes, SARS-CoV-2 infected cultures showed only a slight increment of the expression of *IFN-β* and the expression was reduced in SARS-CoV-2 after RU.521 treatment (Fig. 5 B). In primary rat cortical cultures, the production of type-I interferons such as IFN-β depends on the innate immune response to viruses guided by microglia and astrocytes (Rho et al. 1995). However, an increased expression of *IFN-β* has also been shown to be detrimental, by inducing neuroinflammation and potentially even affecting cognition in humans (Tan et al. 2022). At 24hpi, our results showed a weak *IFN-β* response in rat cortical cells which may lead to a poor immune response. On the other hand, TNF, results showed a higher expression in SARS-CoV-2 infected cultures and a clear inhibitory effect after treatment with RU.521 (Fig. 5 B).

Consistent with the previous results, we observed an increase expression of both chemokines (*CCL2* and *CCL5*) at 24hpi. Also, there was an effective inhibition of *CCL5* but not *CCL2* after RU.521 treatment (Fig. 5 B). Importantly, CCL2 and CCL5 are known to drive inflammatory monocyte infiltration into the brain during viral infection, supporting a key role of these chemokines on inflammation (Ubogu et al. 2006; Howe et al. 2017). Interestingly, rat cortical cultures exposed to SARS-CoV-2 UV-inactivated virus showed an increased expression of the chemokines and cytokines, except for *TNF*, potentially pointing to an immune response elicited by the non-replicative UV-inactivated SARS-CoV-2 still recognized as a pathogenic antigen (Fig. 5 B).

To verify cGAS activation we used a direct cGAS agonist, dsDNA 45 bp (Xia et al. 2019). Primary rat cortical cultures were transfected with dsDNA 45 bp and treated with the inhibitor. Samples were collected to analyze the transcriptional induction of cytokines and chemokines genes at 24 h. As expected, we observed an increase expression of cytokines (*TNF* and *IFN-β*) and chemokines (*CCL2* and *CCL5*) when cells were treated with dsDNA 45 bp, which was inhibited by RU.521 treatment. These results point to a cytokine and chemokine expression profile compatible with what was observed after SARS-CoV-2 infection in human astrocytes and microglia (Fig. 5 C).

Glial cells mediate the initiation and amplification of inflammation in the central nervous system (Ramesh et al. 2013; Yang and Zhou 2019). Viral infection can activate the glial cells present in the rat neocortex, not only inducing the expression but also the production and release of pro-inflammatory cytokines and chemokines. Accordingly, we checked for changes in the production of cytokines and chemokines in the supernatant of primary cortical cultures by enzyme linked immunosorbent assay (ELISA). Since spontaneous spikes and bursts were observed until 6hpi, we used as reference timepoint 6hpi for the mock-infected negative controls (V-), and 6hpi and 24hpi for UV-SARS-CoV-2 control cultures (UV) and SARS-CoV-2-infected cultures (V +).

As shown in Fig. 5 D, we observed that at 6 h the levels of protein production were still low, then, at 24 hpi the concentration of cytokines IFN-γ and TNF increase in the supernatant of SARS-CoV-2 infected cultures. Moreover, there was a statistically significant increment of IFN-γ in the supernatant of infected cells. IFN-γ play an important role in the overall inhibition of neurotropic coronavirus strain JHM infection, in a mouse model of acute viral infection that progresses to a chronic infection (Parra et al. 1999). The production of TNF, another key pro-inflammatory cytokine, was slightly higher at 24hpi in SARS-CoV-2 infected.

Interestingly, we observed an increased production of chemokines CCL2 and CCL5 at 24 hpi in the supernatant of infected cells. These chemokines are involved in acute inflammation and are related to the recruitment of monocytes and polymorphonuclear cells and the persistent maintenance of inflammation (Hussmann and Fredericksen 2014; Howe et al. 2017; Mladinich et al. 2021). This increase in chemokines thus seems to confirm the immune activation of cortical cultures after SARS-CoV-2 infection.

Overall, SARS-CoV-2 infection induced not only the expression but also the production of pro-inflammatory cytokines and chemokines. Activation of cGAS-STING pathway initiates a pro-inflammatory response with the purpose of fighting the infection and repairing the damage. In our model may also be associated to excessive release of pro-inflammatory factors, recruit of neutrophils and macrophages, that contribute to neuroinflammation leading to the loss of electrical activity which in turns may lead to tissue damage, neurodegeneration and neuronal loss.

### cGAS inhibitor RU.521 rescues the electrical activity of infected rat cortical cultures in the first six hours post-infection

To study the effect of the cGAS-STING pathway on the electrical activity of SARS-CoV-2 infected cultures, we performed MEA recordings in the presence of a cGAS inhibitor, RU.521 (Vincent et al. 2017). As for the previous electrophysiology experiments, n = 12 cultures of rat cortical neurons were plated and kept for 21 days in vitro. For every experiment, one SARS-CoV-2-infected culture and one UV-SARS-CoV-2 control were recorded in parallel for 24 h. The electrical activity was recorded before infection with SARS-CoV-2, then the cultures were infected and treated after 2 h with 10 μM of the RU.521 cGAS inhibitor, after which recordings were performed at three time points: right after the treatment with RU.521 (i.e. 2 hpi), after 3 h and after 6 h.

**Fig. 6:**
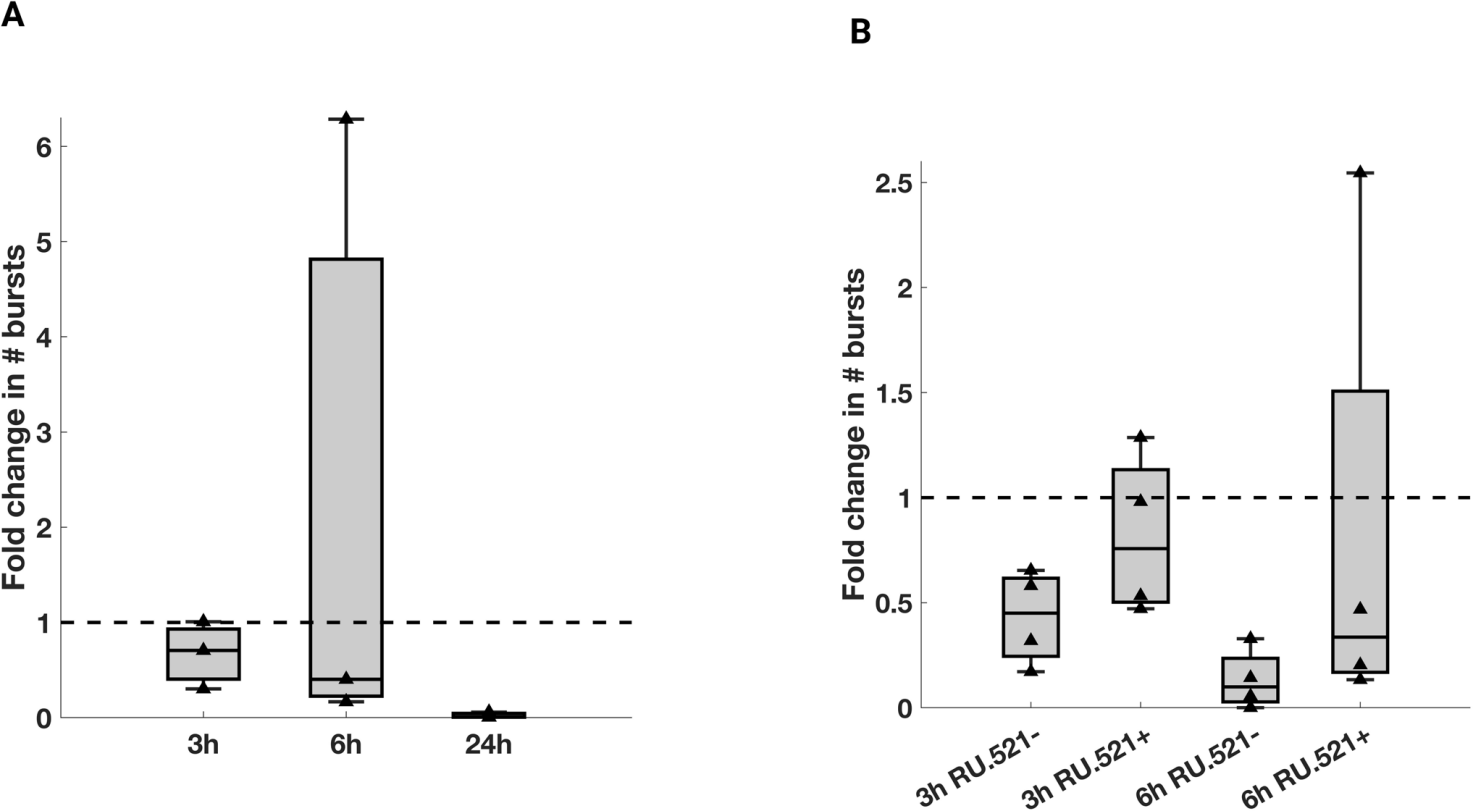
**Electric activity in SARS-CoV-2 infected rat cortical cells is rescued by the cGAS inhibitor RU.521**. **A** Fold change in the number of bursts after incubation with RU.521 cGAS-STING inhibitor. The plot shows the number of bursts recorded from n = 3 MEAs incubated for 2 h with RU.521 and normalized by the number of bursts recorded before incubation (bi) in the same MEA; no statistically significant difference was found *(Friedman test, χ*^*2*^*(3)* = *5.80, p* = *1.22* × *10*^*–1*^*)*. **B** Fold change in the number of bursts in MEAs infected with SARS-CoV-2, with and without RU.521. Number of bursts recorded from n = 4 MEAs infected with SARS-CoV-2, and n = 4 MEAs infected with SARS-CoV-2 and treated with RU.521. The number of bursts for each timepoint is normalized by the number of bursts recorded before incubation (bi) in the same MEA; no statistically significant difference was found *(Friedman test, χ*^*2*^*(3)* = *6.30, p* = *9.79* × *10*^*–2*^*)*

In both SARS-CoV-2-infected and UV-SARS-CoV-2 control cultures treated with RU.521 we did not observe activity at 24 hpi (data not shown). To establish the effect of RU.521 alone, we performed a series of recordings on n = 3 MEAs incubating them with RU.521 for 2 h (without SARS-CoV-2 infection). Although the difference was not statistically significant *(Friedman test, χ*^*2*^*(3)* = *5.80, P* = *1.22* × *10*^*–1*^*)*, the number of bursts decreased dramatically from 495 ± 287.3 before incubation (bi) to 5 ± 19.5 at 24 h (median ± IQR, Fig. 6 A) suggesting that RU.521 alone can impair electrical activity at this timepoint. The lack of statistical significance may be due to the small sample size.

For this reason, we decided to focus our attention on early timepoints, and we compared the results obtained in cultures infected with SARS-CoV-2 and then incubated with RU.521 with recordings performed without the cGAS inhibitor (N = 4 MEAs). As shown in Fig. 6 B, the number of bursts decreased over time for both conditions. However, the reduction was more pronounced in cultures not treated with RU.521 at 3 h and 6 h. Also in this case, the difference was not statistically significant, possibly due to the small sample size *(Friedman test, χ*^*2*^*(3)* = *6.30, p* = *9.79* × *10*^*–2*^*).*

These point out a possible rescue effect of the cGAS inhibitor RU.521 in the first hours after infection, which seems to be able to reduce the inflammatory effect activated by SARS-CoV-2 infection.

## Discussion

Post-COVID-19 symptoms vary from 12 to 45% of patients (Ballering et al. 2022; O’Mahoney et al. 2023). Some of the mild symptoms that are associated to the CNS are headache, brain fog, memory loss and insomnia (Canas et al. 2023), but severe symptoms such as stroke, cerebral thrombosis, seizures, meningoencephalitis, Guillain-Barré syndrome have been described (rev in (Harapan and Yoo 2021)). Notwithstanding the great concern caused by these clinical cases, the neuropathogenicity of SARS-CoV-2 infection still remains largely unexplored.

Here we demonstrate that the immune response triggered by glial cells plays a crucial role during SARS-CoV-2 infection of the brain. Although only astrocytes permit effective infection, both iPSC astrocytes and HMC3 microglia showed signs of senescence, recruitment of foci compatible with DNA damage accumulation and activation of the cGAS-STING pathway. Moreover, using rat cortical cells seeded on MEA’s, we identified that SARS-CoV-2 infection decreased the number of network-wide bursts through a decreased synaptic connection, and that this effect could be partially rescued by treating the culture with an inhibitor of cGAS-STING pathway.

Knowing the involvement of glial cells in the response to viral infections (Hwang and Bergmann 2018), we infected both iPSC astrocytes and HMC3 microglia. SARS-CoV-2 infection of glia cells has been detected in vitro, in vivo (Andrews et al. 2022; Jeong et al. 2022; Colinet et al. 2024) and in autopsies of COVID patients (Crunfli et al. 2022). In accordance with previous studies, we observed a productive infection in iPSC astrocytes and an abortive infection in HMC3 microglia (Crunfli et al. 2022; Haverty et al. 2024). Infection of astrocytes with WT SARS-CoV-2 has been demonstrated in iPSC derived brain organoids (Andrews et al. 2022; Kong et al. 2022), which is in agreement with our observation. However, microglial cells are absent in this brain model, therefore tropism for this cell type was not addressed. One study tested different variants of SARS-CoV-2 both in primary astrocytes and HMC3 microglia (Proust et al. 2023). Unlike our results, productive infection was observed in microglia and not in astrocytes. However, we used different viral isolates from the one used by Proust et al. (Proust et al. 2023).

We further investigated the expression of *ACE2* an *TMPRSS2* mRNA levels both in human astrocytes and microglia. We observed a higher expression of both *ACE2* and *TMPRSS2* in astrocytes compared to microglia at basal levels, while SARS-CoV-2 infection increased the expression of *ACE2 *in astrocytes but not in microglia. Interestingly, treatment with the cGAS inhibitor G140 did not change the expression of viral receptors following infection. We also calculated the number of genomic copies of virus per microliter in cell lysates and there was an increment of 2 log after infection in astrocytes, no differences were observed in microglia. Moderate to low presence of both *ACE2* and *TMPRRS2* has been reported in different zones of the CNS (Malik et al. 2023; Gupta et al. 2024; Haverty et al. 2024; Wälchli et al. 2024). In agreement to our findings, primary human astrocytes showed a modest increase in the expression of SARS-CoV-2 host cell entry factors (ACE2, TMPRSS2, NRP1, and TRIM28) (Acharya et al. 2023). Moreover, transcriptomic data from Wälchli et al. showed a similar expression of *ACE2* and* TMPRSS2* in astrocytes (medium expression), while in microglia the expression of the receptors remained low (Wälchli et al. 2024).

The infection of glial cells was accompanied by DNA damage accumulation and an increment of the markers of cellular senescence. We explored the DNA damage repair (Victor et al. 2021; Grand 2023) response in glial cells and found a consistent increase of infected cells showing γH2AX foci in the nucleus. Intriguingly, despite HMC3 microglial cells are poorly infected, we still observed an increase of γH2AX foci.

DNA damage accumulation is commonly observed in the CNS in neurodegenerative diseases associated with ageing (López-Otín et al. 2023), yet little is known in virus related pathogenesis. In astrocytes and microglia, DNA damage contributes to neuroinflammation and toxicity in the context of neurodegenerative diseases (Kok et al. 2021; Talbot et al. 2024). Neurons are extremely sensitive to DNA damage and additionally DNA damage in glial cells may induce chronic inflammation that leads to neurotoxicity. When corrupted, DNA is mislocated in the cytoplasm and the cytosolic DNA sensor cGAS initiates a signaling pathway with a potent inflammatory effect.

Both human glial cell types studied here can sense cytosolic DNA, express cGAS and its downstream adaptor molecule STING (Jeffries and Marriott 2017), and induce cGAS-STING dependent cytokine and chemokine production (Jeffries and Marriott 2017; Talbot et al. 2024). In accordance with previous studies, we observed that glial cells may play a crucial role in maintaining the inflammatory environment of the CNS after infection (rev in (Giovannoni and Quintana 2020)). In particular, the increase of IFN-β is evidence of the role of astrocytes during SARS-CoV-2 infection and may be associated with the recruitment of additional glial cells, following a well-known pathway of viral infection response (Clarke et al. 2019). Moreover, the significant action of astrocytes as IFN-β producers after acute neurotropic infections in the CNS is well described (Pfefferkorn et al. 2016). However, it was previously demonstrated that inflammation of the CNS directed by SARS-CoV-2 infection of astrocytes promotes neuronal dysfunction and cellular death (Kong et al. 2022), mainly mediated by exacerbated interferon response. Consistently, our study shows a marked induction of IFN-β response of iPSC astrocytes after SARS-CoV-2 that was inhibited after treatment with an inhibitor of cGAS. We also observed that HMC3 reaction to SARS-CoV-2 was consistent with literature on the role of microglia in response to viral infection (Filgueira et al. 2021). In agreement with previous studies, microglia is poorly infected by SARS-CoV-2 but shows an upregulation of genes related to microglia activation, cytokine secretion and inflammation (Andrews et al. 2022). Neuroinflammation is a response of CNS cells as neurons, astrocytes and microglia to different stimuli, and it is mainly mediated by cytokines and chemokines (rev in (Shabab et al. 2017)). Activation of astrocytes and microglia not only initiate but also amplify the inflammatory response in the CNS. Importantly, we detected a higher number of micronuclei positive to cGAS in both glial cell types and a general inhibition of the expression of the cytokines and chemokines when a cGAS inhibitor was added to the culture, meaning that after SARS-CoV-2 infection, the cGAS-STING pathway plays an important role in the pro-inflammatory response of glial cells. Those findings agree with another in vitro study, where SARS-CoV-2 Calu3 infected cells (Gioia et al. 2023) exhibited higher number of micronuclei positive to cGAS and higher transcription induction of pro-inflammatory cytokines. Moreover, studies on human samples showed that cGAS-STING pathway participates in the response of endothelial cells towards SARS-CoV-2 infection as well as in driving type I IFN responses in COVID-19 skin lesions (Domizio et al. 2022). Moreover, postmortem lung tissue exhibited higher expression of active STING in tissues from patients with lethal disease outcome (Domizio et al. 2022). Blood samples of acute infection and long COVID patients showed elevated *cGAS*, *STING* and *IFN-α* levels, which was directly associated to long COVID, most likely contributing to systemic inflammation in severe patients (Queiroz et al. 2024).

The inflammatory response depends on multiple transcription factors. STING activation recruits TBK1 which in turn triggers other downstream pathways such as NF-κB and IRF3. Therefore, we next studied the activation of the cGAS–STING–NF-κB axis in glial cells. We analysed at a protein level the total and phosphorylated forms of TBK1 and NF-κB in infected cells treated with a cGAS inhibitor. We observed that infection induced an increment of total protein detection both in iPSC astrocytes and HMC3 microglia. Activation of TBK1 and NF-κB in infected cells was not evident, except for a slight activation of NF-κB in HMC3. Higher activation of NF-κB has been observed in severe cases of COVID (Kircheis et al. 2020). In the brain, one study evaluated RNAseq data from human cortex after SARS-CoV-2 infection revealing an inflammatory response from activated astrocytes with high NF-κB signalling (Andrews et al. 2022). Although NF-κB activation may increase the response against SARS-CoV-2, the dysregulated inflammatory response could markedly intensify the symptoms as reported in lungs (Neufeldt et al. 2022). Downstream signaling components such as TBK1 and NF-κB are partake by RNA and DNA sensing pathways. Well known antiviral responses are activated after cytosolic viral RNA is recognize by the Retinoic acid inducible gene-I (RIG-I) receptor and TLR’s, that further prompt the expression of pro-inflammatory genes via the TBK1-NF-κB axis. The role of RIG-I during SARS-CoV-2 infection was demonstrated in vivo (Marx et al. 2022) and in vitro (Thorne et al. 2021). Since we previously reported the significance of the DNA damage response and cGAS-STING pathway (Gioia et al. 2023), we focused our work on the role of the DNA sensing pathway in the brain. Indeed, we found activation of NF-κB in infected microglia; therefore, dsRNA needed for the activation of RNA sensing pathways, is rarely produced when virus replication is scares or absent. Moreover, since we observe damage accumulation in glial cells, cytosolic DNA may be sensed by cGAS starting the pro-inflammatory cascade independently from the magnitude of virus replication. Another distinct aspect of the pathway is that cGAMP (Ablasser et al. 2013) can move between infected and bystander cells, allowing uninfected cells to propagate antiviral cytokine responses through STING activation. We are aware that in RNA and DNA sensing pathways the downstream signaling elements are interconnected. Therefore, lack of evidence of the contribution of RNA sensing pathway to the inflammatory signature presented here opens the possibility to further investigations. Next, we investigated if human glial cells may suffer cellular stress associated to SARS-CoV-2 infection. We identified cellular senescence and DNA damage accumulation in both astrocytes and microglia exposed to the virus. Previous studies described virus induced senescence (VIS) induced by SARS-CoV-2 showing increased levels of p21 staining (Lee et al. 2021; Evangelou et al. 2022; Gioia et al. 2023; Delval et al. 2023). VIS have been described in primary cells, in vivo (Lee et al. 2021; Gioia et al. 2023) and in autopsies (Lee et al. 2021; Evangelou et al. 2022; Gioia et al. 2023) of the respiratory tract of COVID-19 patients, yet in the CNS this information is lacking. Importantly, senescence in astrocytes has been correlated to proliferation arrest and activation of pro-inflammatory responses, leading to the development of neurodegenerative diseases such as Alzheimer’s disease (AD) (He et al. 2013) and Parkinson’s disease (PD) (rev in (Hernandez et al. 2016)). Moreover, the increment of senescent microglia, negatively modified its immune functions and the interactions with other brain cells, which in turn collaborated to the progress of the pathogenesis of neurodegenerative conditions (rev in (Angelova and Brown 2019)). Therefore, the spreading of cellular senescence to neighbouring cells can contribute to neuroinflammation, inducing the phenomena referred as senescence-associated secretory phenotype (SASP).

Once glial cells were identified as key players involved in the immune response against SARS-CoV-2, we explored how the presence of cell senescence, DNA damage accumulation and pro-inflammatory responses might affect neurons in the context of a cortical cell culture. Astrocytes are key players for maintaining the CNS homeostasis; by closely interacting with neurons, they modulate the synapse formation as well as function and maintaining of ion and neurotransmitter concentrations (rev in (Melo Dos Santos et al. 2024)). However, reactive astrocytes showing a pro-inflammatory profile may contribute to neuronal degeneration.

In our study we aimed at investigating the molecular pathways of inflammation in the CNS following direct SARS-CoV-2 infection. To this end, we introduced a novel ex vivo rat cortical culture model to study the electrophysiology of the infection. Lack of an in vivo model of infection is a limit of our work. However, only few animal models are currently available to study the neurological symptoms observed in COVID patients. The larger evidence are studies with mice carrying the hACE2 sequence under the control of the cytokeratin-18 (K18) gene promoter (K18-hACE2-tg) (Moreau et al. 2020; Song et al. 2021; Fumagalli et al. 2022), which induces widespread expression of the receptor irrespective of the cell type. Intranasal inoculation leads to massive neuroinvasion and neuroinflammation, activation of astrocytes and microglia together with neurological symptoms that can be fatal (Dong et al. 2022). Aerosol inoculation of the virus leads to a milder outcome (Fumagalli et al. 2022). Another mice model is the hACE2-knock in (KI)—expression of the human receptor is under the control of the endogenous promoter of mACE2—the animals presented low amounts of viral RNA detected in brain samples, but the studies are limited to the acute phase (Zhou et al. 2021; Winkler et al. 2022). The Golden Syrian hamsters are naturally infected with SARS-CoV-2 and develop neurological manifestations as accumulation of hyper-phosphorylated tau and α-synuclein in cortical neurons, transcriptomic perturbations in the CNS and behavioural changes (Käufer et al. 2022; Frere et al. 2022; Carpenter et al. 2023). Finally, non-human primates are naturally susceptible to SARS-CoV-2 infection and present signs of neuroinflammation without evidence of neuroinvasion (revised by (Usai et al. 2023)). Recently, a preprint article claimed that after infection with a mouse adapted SARS-CoV-2, WT C57BL/6 J mice developed persisting neurological damage, behaviour changes and inflammation in several organs including the brain (Qiao et al. 2024). Since human in vivo electrophysiological studies are hardly practicable, *in vitro *cortical cultures have become an important system model to study virus neuropathogenesis. Here we explored the capacity of rat cortical culture as a suitable model to study SARS-CoV-2 infection in the brain context. Primary rat cortical cultures are widely used to study neuroinflammation (Goshi et al. 2020) and neurodegeneration, to investigate in vitro the electrophysiology (Dégenètais et al. 2002; Goshi et al. 2023) of neural networks. This approach ensures that the appropriate cell types are included and recapitulates the in vivo homeostasis and crosstalk within neurons and glia.

The study of WT (Zhang et al. 2022) and humanized ACE2 (Jiang et al. 2022) rat as model for SARS-CoV-2 infection is rarely represented (Bosco-Lauth et al. 2021), probably due to the higher cost of maintenance compared to mouse. Also, only a few studies investigated the tropism of the virus in wild rats (Miot et al. 2022; Colombo et al. 2022; Wang et al. 2023). To the best of our knowledge, we have shown for the first time that SARS-CoV-2 is capable of infecting WT primary rat cortical cultures. While rodent models expressing human ACE2 have been developed since the inception of the COVID-19 pandemic (Israelow et al. 2020; Rathnasinghe et al. 2020), being able to use WT animals increases the accessibility and therefore the effort to understand the SARS-CoV-2 neuropathogenesis. Moreover, maintenance of in vitro electrophysiology activity of rat cortical culture is fundamental to study virus-induced inflammation and cell survival. SARS-CoV-2 was found to be able to infect both neurons and glial cells, with a clear prevalence for glial cells. This result is consistent with findings in human organoids (McMahon et al. 2021) and both adult and developing human organotypic studies (Andrews et al. 2022).

The effect of virus infection in neurophysiology has been poorly studied. One study using in vitro embryonic mouse primary neuron cultures infected with Zika virus (Gaburro et al. 2018), determined that the infection activates an early excitatory response that was followed by a dramatic loss of this activity, similar to our observations. We recorded the electrical activity of primary cultures seeded on MEAs at different timepoints after infection. We found that the overall activity (single-unit spikes and population-wide bursts) decreased with the progression of SARS-CoV-2 infection. Specifically, the frequency and the amplitude of bursts decreased in time. With a stimulation protocol, we were able to show that the immediate effect of the infection is the loss of synaptic connectivity, represented by recurrent synaptic firing, and only at later timepoints we observed the loss of antidromic response, signaling neuronal loss. In agreement, one study observed synaptic loss in neurons when human brain organoids were infected with SARS-CoV-2. Sofosbuvir was able to inhibit SARS-CoV-2 replication and rescued these neuronal alterations (Mesci et al. 2022). Indeed, we observed a reduction in the number and size of the Synapsin 1 marker of synapses, meaning that the integrity of the synaptic vesicles was affected after infection. In agreement with MEA findings, loss of synaptic vesicles may indicate an impairment of the neurophysiological activity and the synaptic connections. In a rat model of Alzheimer’s disease (AD) overexpression of Synapsin 1 was correlated to an improvement of cognitive function together with increase release of neurotransmitters and inhibition of the inflammatory responses (Ma et al. 2023). Synaptic markers were also reduced after infection with human immunodeficiency virus (HIV) (Gelman and Nguyen 2010) and Herpes simplex virus (Piacentini et al. 2015).

Being glial cells highly susceptible to SARS-CoV-2, we became interested in understanding how infection may induce glial activation that in turn affects the electrical activity in vitro. It is well known that astrocytes contribute to synaptic activity in the CNS (Perea et al. 2009), and they are also involved in the inflammatory reaction to viral infection (Jorgačevski and Potokar 2023). Thus, we first analyzed the expression levels of cytokines and chemokines in rat cortical cultures. SARS-CoV-2 infection increased the production of pro-inflammatory factors, which could potentially lead to the loss of synaptic connectivity and, at a later timepoint, to neuronal death. Indeed, cytokines have been shown to modulate synaptic connectivity and neuronal function (Werneburg et al. 2017; Matelski et al. 2021). As hypothesized, electrophysiological activity was significantly reduced after infection. However, a recent study observed that microglia was able to initiate a pro-inflammatory response after treatment with both UV-inactivated virus and Spike protein alone (Clough et al. 2021). To confirm the relationship to productive infection, we used UV-inactivated virus, which caused only a marginal effect on cytokine production and electrophysiological activity, while purified Spike protein was completely inactive.

In the context of neuroinflammation, we hypothesized that the cGAS–STING signaling pathway may be a key mediator of inflammation in the CNS upon SARS-CoV-2 infection, as observed in neurodegenerative Parkinson’s disease (Sliter et al. 2018). In a previous work, it was demonstrated that the pro-inflammatory cGAS-STING pathway was activated by the presence of endogenous damaged DNA during SARS-CoV-2 infection (Gioia et al. 2023). Therefore, we checked for the presence of markers of DDR and found that after infection, foci of DNA damage were more likely to be present in glial cells in the infected primary cortical cultures. To confirm the involvement of the cGAS-STING pathway, we tested the RNA expression of pro-inflammatory cytokines in the presence of antagonists for the pathway itself. Inhibition of cGAS inhibited the induction of cytokines and chemokines following SARS-CoV-2 both in human and rat cells.

SARS-CoV-2 infection increased the production of pro-inflammatory factors in the supernatants of infected cultures, which could potentially lead to the loss of synaptic connectivity and, at a later timepoint, to neuronal death. Indeed, cytokines have been shown in literature to modulate synaptic connectivity and neuronal function (Werneburg et al. 2017; Matelski et al. 2021).This finding is compatible with the activation of the cGAS-STING pathway and is consistent with the response of the pathway to infection. In fact, activation of cGAS-STING pathway after SARS-CoV-2 infection was confirmed in several studies as a key mediator of the inflammatory response and antiviral responses (Domizio et al. 2022; Puray-Chavez et al. 2024). Finally, we returned to electrophysiology to confirm that the cGAS-STING pathway was indeed involved in the synaptic loss at early stages of SARS-CoV-2 infection from a functional point of view. To the best of our knowledge, this study is the first to use MEAs to test changes in electrophysiology related to the activation of the cGAS-STING pathway. We incubated infected primary cortical cultures seeded on MEAs with the cGAS antagonist RU.521. While higher levels of antagonist in primary cortical cultures were able to better reduce the pathogenic effects of infection, we also found them to decrease electrical activity per se. However, we found that the number of network-wide bursts in cultures treated with the cGAS-STING antagonist was indeed higher, pointing to a rescue effect when inhibiting the cGAS-STING pathway.

To conclude, we provide evidence for a senescence phenotype and cGAS-dependent inflammatory response in the brain cortex when infected by SARS-CoV-2 ex vivo. While a limit of the study is the still debated notion of direct SARS-CoV-2 infection of the brain of infected individuals, targeting this pathway with specific senolytics could provide a potential pathway for the treatment of long-term consequences of COVID-19.

## Supplementary Information

Below is the link to the electronic supplementary material.

Supplementary file1 (PDF 3045 KB)

Supplementary file2 (PDF 1371 KB)

Supplementary file3 (PDF 1886 KB)

Supplementary file4 (PDF 2440 KB)

Supplementary file5 (PDF 216 KB)

## Data Availability

The raw data that support the findings of this study are available from the corresponding author, MG and AM, upon reasonable request.

## Abbreviations

AD: Alzheimer’s disease
ACE2: Angiotensin-converting enzyme 2
BSA: Bovine serum albumin fraction V
CMC: Carboxymethylcellulose
CNS: Central nervous system
COVID: Coronavirus disease
DDR: DNA damage response
ELISA: Enzyme linked immunosorbent assay
GABA: Gamma-aminobutyric acid
GFAP: Glial fibrillary acid protein
GAPDH: Glyceraldehyde-3-phosphate dehydrogenase
cGAMP: Guanosine monophosphate–adenosine monophosphate
HIV: Human immunodeficiency virus
HMC3: Human microglial clone 3
IF: Immunofluorescence
iPSC: Induced pluripotent stem cells
IFNs: Interferons
IBA1: Ionized calcium-binding adapter molecule 1
MEAs: Microelectrode arrays
MOI: Multiplicity of infection
NPCs: Neural progenitor cells
NfL: Neurofilament light chain protein
NMDA: N-methyl D-aspartate
N: Nucleocapsid protein
PD: Parkinson’s disease
PBS: Phosphate-buffered saline
PFU/mL: Plaque forming units per milliliter
qRT-PCR: Real-time quantitative reverse transcription PCR
SASP: Senescence-associated secretory phenotype
SARS-CoV-2: Severe Acute Respiratory Syndrome Coronavirus-2
S: Spike
VID: Virus induced DNA damage
VIS: Virus induced senescence
WB: Western blot
WT: Wild type

## References

[CR1] Ablasser A, Schmid-Burgk JL, Hemmerling I et al (2013) Cell intrinsic immunity spreads to bystander cells via the intercellular transfer of cGAMP. Nature 503:530–534. 10.1038/nature12640 24077100 10.1038/nature12640 PMC4142317

[CR2] Acharya A, Ambikan A, Thurman M, et al (2023) Proteomic landscape of astrocytes and pericytes infected with HIV/SARS-CoV-2 mono/co-infection, impacting on neurological complications. Res Sq. 10.21203/rs.3.rs-3031591/v1

[CR3] Albornoz EA, Amarilla AA, Modhiran N (2023) SARS-CoV-2 drives NLRP3 inflammasome activation in human microglia through spike protein. Mol Psychiatry 28(7):2878–2893. 10.1038/s41380-022-01831-0 36316366 10.1038/s41380-022-01831-0 PMC10615762

[CR4] Andrews MG, Mukhtar T, Eze UC et al (2022) Tropism of SARS-CoV-2 for human cortical astrocytes. Proc Natl Acad Sci USA 119:1–12. 10.1073/pnas.2122236119 10.1073/pnas.2122236119 PMC9335272 35858406

[CR5] Angelova DM, Brown DR (2019) Microglia and the aging brain: are senescent microglia the key to neurodegeneration? J Neurochem 151:676–688. 10.1111/jnc.14860 31478208 10.1111/jnc.14860

[CR6] Antony AR, Haneef Z (2020) Systematic review of EEG findings in 617 patients diagnosed with COVID-19. Seizure 83:234–241. 10.1016/j.seizure.2020.10.014 33121875 10.1016/j.seizure.2020.10.014 PMC7569418

[CR7] Balka KR, Louis C, Saunders TL (2020) TBK1 and IKKε act redundantly to mediate STING-induced NF-κB responses in myeloid cells. Cell Rep 31(1):107492. 10.1016/j.celrep.2020.03.056 32268090 10.1016/j.celrep.2020.03.056

[CR8] Ballering AV, van Zon SKR, Olde Hartman TC, Rosmalen JGM (2022) Persistence of somatic symptoms after COVID-19 in the Netherlands: an observational cohort study. Lancet 400:452–461. 10.1016/S0140-6736(22)01214-4 35934007 10.1016/S0140-6736(22)01214-4 PMC9352274

[CR9] Blau, Axel, Tanja Neumann, Christiane Ziegler, and Fabio Benfenati (2009) “Replica-moulded polydimethylsiloxane culture vessel lids attenuate osmotic drift in long-term cell cultures.” Springer Science; Business Media LLC. J Biosci 34(1):59–69. 10.1007/s12038-009-0009-3 10.1007/s12038-009-0009-3 19430119

[CR10] Bonifazi P, Goldin M, Picardo MA et al (2009) GABAergic hub neurons orchestrate synchrony in developing hippocampal networks. Science 326:1419–1424. 10.1126/science.1175509 19965761 10.1126/science.1175509

[CR11] Bosco-Lauth AM, Root JJ, Porter SM et al (2021) Peridomestic mammal susceptibility to severe acute respiratory syndrome coronavirus 2 infection. Emerg Infect Dis 27:2073–2080. 10.3201/eid2708.210180 34286685 10.3201/eid2708.210180 PMC8314817

[CR12] Butowt R, Meunier N, Bryche B, Bartheld CS (2021) The olfactory nerve is not a likely route to brain infection in COVID - 19 : a critical review of data from humans and animal models. Acta Neuropathol 141:809–822. 10.1007/s00401-021-02314-2 33903954 10.1007/s00401-021-02314-2 PMC8075028

[CR13] Canas LS, Molteni E, Deng J et al (2023) Profiling post-COVID-19 condition across different variants of SARS-CoV-2: a prospective longitudinal study in unvaccinated wild-type, unvaccinated alpha-variant, and vaccinated delta-variant populations. Lancet Digit Health 5:e421–e434. 10.1016/S2589-7500(23)00056-0 37202336 10.1016/S2589-7500(23)00056-0 PMC10187990

[CR14] Carpenter KC, Yang J, Xu JJ (2023) Animal models for the study of neurologic manifestations of COVID-19. Comp Med 73(1):91–103. 10.30802/AALAS-CM-22-000073 36744556 10.30802/AALAS-CM-22-000073 PMC9948905

[CR15] Chen Z, Li G (2021) Immune response and blood–brain barrier dysfunction during viral neuroinvasion. Innate Immun 27:109–117. 10.1177/1753425920954281 32903111 10.1177/1753425920954281 PMC7882805

[CR16] Clarke P, Zhuang Y, Berens HM (2019) Interferon beta contributes to astrocyte activation in the brain following reovirus infection. J Virol. 10.1128/JVI.02027-18 30814290 10.1128/JVI.02027-18 PMC6498044

[CR17] Clough E, Inigo J, Chandra D et al (2021) Mitochondrial dynamics in SARS-COV2 spike protein treated human microglia: implications for neuro-COVID. J Neuroimmune Pharmacol 16:770–784. 10.1007/s11481-021-10015-6 34599743 10.1007/s11481-021-10015-6 PMC8487226

[CR18] Colinet M, Chiver I, Bonafina A, et al (2024) SARS-CoV2 infection triggers reactive astrocyte states and inflammatory conditions in long-term Human Cortical Organoids. bioRxiv 2024.04.16.589036 10.1093/stmcls/sxaf010 PMC12121356 40103011

[CR19] Colombo VC, Sluydts V, Mariën J et al (2022) SARS-CoV-2 surveillance in Norway rats (Rattus norvegicus) from Antwerp sewer system, Belgium. Transbound Emerg Dis 69:3016–3021. 10.1111/tbed.14219 34224205 10.1111/tbed.14219 PMC8447303

[CR20] Crunfli F, Carregari VC, Veras FP, et al (2022) Morphological , cellular , and molecular basis of brain infection in COVID-19 patients. 0:1–12 10.1073/pnas.2200960119 PMC9436354 35951647

[CR21] Croitoru-Lamoury J, Guillemin GJ, Boussin FD et al (2003) Expression of chemokines and their receptors in human and simian astrocytes: evidence for a central role of TNF alpha and IFN gamma in CXCR4 and CCR5 modulation. Glia 41:354–370. 10.1002/glia.10181 12555203 10.1002/glia.10181

[CR22] Darif D, Hammi I, Kihel A et al (2021) The pro-inflammatory cytokines in COVID-19 pathogenesis : what goes wrong ? Microb Pathog. 10.1016/j.micpath.2021.104799 33609650 10.1016/j.micpath.2021.104799 PMC7889464

[CR23] De Camilli P, Harris SMJ, Huttner WB, Greengard P (1983) Synapsin I (protein I), a nerve terminal-specific phosphoprotein. II. Its specific association with synaptic vesicles demonstrated by immunocytochemistry in agarose-embedded synaptosomes. J Cell Biol 96:1355–1373. 10.1083/jcb.96.5.1355 6404911 10.1083/jcb.96.5.1355 PMC2112643

[CR24] Decout A, Katz JD, Venkatraman S, Ablasser A (2021) The cGAS–STING pathway as a therapeutic target in inflammatory diseases. Nat Rev Immunol 21:548–569. 10.1038/s41577-021-00524-z 33833439 10.1038/s41577-021-00524-z PMC8029610

[CR25] Dégenètais E, Thierry A-M, Glowinski J, Gioanni Y (2002) Electrophysiological properties of pyramidal neurons in the rat prefrontal cortex: an in vivo intracellular recording study. Cereb Cortex 12:1–16. 10.1093/cercor/12.1.1 11734528 10.1093/cercor/12.1.1

[CR26] Delval L, Hantute-Ghesquier A, Sencio V et al (2023) Removal of senescent cells reduces the viral load and attenuates pulmonary and systemic inflammation in SARS-CoV-2-infected, aged hamsters. Nat Aging 3:829–845. 10.1038/s43587-023-00442-w 37414987 10.1038/s43587-023-00442-w PMC10353934

[CR27] Domizio JD, Gulen MF, Saidoune F (2022) The cGAS-STING pathway drives type i IFN immunopathology in COVID-19. Nature 603:145–151. 10.1038/s41586-022-04421-w 35045565 10.1038/s41586-022-04421-w PMC8891013

[CR28] Domm S, Cinatl J, Mrowietz U (2008) The impact of treatment with tumour necrosis factor-α antagonists on the course of chronic viral infections: a review of the literature. Br J Dermatol 159:1217–1228. 10.1111/j.1365-2133.2008.08851.x 18945310 10.1111/j.1365-2133.2008.08851.x

[CR29] Dong W, Mead H, Tian L et al (2022) The K18-human ACE2 transgenic mouse model recapitulates non-severe and severe COVID-19 in response to an infectious dose of the SARS-CoV-2 virus. J Virol 96:e00964–21. 10.1128/JVI.00964-21 34668775 10.1128/JVI.00964-21 PMC8754221

[CR30] Evangelou K, Veroutis D, Paschalaki K et al (2022) Pulmonary infection by SARS-CoV-2 induces senescence accompanied by an inflammatory phenotype in severe COVID-19: possible implications for viral mutagenesis. Eur Respir J. 10.1183/13993003.02951-2021 35086840 10.1183/13993003.02951-2021 PMC8796696

[CR31] Fang R, Wang C, Jiang Q et al (2017) NEMO-IKKβ are essential for IRF3 and NF-κB activation in the cGAS-STING pathway. J Immunol 199:3222–3233. 10.4049/jimmunol.1700699 28939760 10.4049/jimmunol.1700699

[CR32] Fara A, Mitrev Z, Rosalia RA, Assas BM (2020) Cytokine storm and COVID-19: a chronicle of pro-inflammatory cytokines: cytokine storm: the elements of rage! Open Biol 10:10.1098/rsob.200160 10.1098/rsob.200160 PMC7536084 32961074

[CR33] Filgueira L, Larionov A, Lannes N (2021) The influence of virus infection on microglia and accelerated brain aging. Cells. 10.3390/cells10071836 34360004 10.3390/cells10071836 PMC8303900

[CR34] Frere JJ, Serafini RA, Pryce KD et al (2022) SARS-CoV-2 infection in hamsters and humans results in lasting and unique systemic perturbations after recovery. Sci Transl Med 14:eabq3059. 10.1126/scitranslmed.abq3059 35857629 10.1126/scitranslmed.abq3059 PMC9210449

[CR35] Fumagalli V, Rava M, Marotta D (2022) Administration of aerosolized SARS-CoV-2 to K18-hACE2 mice uncouples respiratory infection from fatal neuroinvasion. Sci Immunol. 10.1126/sciimmunol.abl9929 34812647 10.1126/sciimmunol.abl9929 PMC9835999

[CR36] Gaburro J, Bhatti A, Sundaramoorthy V et al (2018) Zika virus-induced hyper excitation precedes death of mouse primary neuron. Virol J 15:79. 10.1186/s12985-018-0989-4 29703263 10.1186/s12985-018-0989-4 PMC5922018

[CR37] Gelman BB, Nguyen TP (2010) Synaptic proteins linked to HIV-1 infection and immunoproteasome induction: proteomic analysis of human synaptosomes. J Neuroimmune Pharmacol 5:92–102. 10.1007/s11481-009-9168-0 19693676 10.1007/s11481-009-9168-0 PMC2824116

[CR38] Gerber L-S, van Melis LVJ, van Kleef RGDM et al (2021) Culture of Rat Primary Cortical Cells for Microelectrode Array (MEA) Recordings to Screen for Acute and Developmental Neurotoxicity. Curr Protoc 1:e158. 10.1002/cpz1.158 34152700 10.1002/cpz1.158

[CR39] Gioia U, Tavella S, Martínez-Orellana P et al (2023) SARS-CoV-2 infection induces DNA damage, through CHK1 degradation and impaired 53BP1 recruitment, and cellular senescence. Nat Cell Biol. 10.1038/s41556-023-01096-x 36894671 10.1038/s41556-023-01096-x PMC10104783

[CR40] Giovannoni F, Quintana FJ (2020) The role of astrocytes in CNS inflammation. Trends Immunol 41:805–819. 10.1016/j.it.2020.07.007 32800705 10.1016/j.it.2020.07.007 PMC8284746

[CR41] Giugliano M, Darbon P, Arsiero M et al (2004) Single-neuron discharge properties and network activity in dissociated cultures of neocortex. J Neurophysiol 92:977–996. 10.1152/jn.00067.2004 15044515 10.1152/jn.00067.2004

[CR42] Goshi N, Morgan RK, Lein PJ, Seker E (2020) A primary neural cell culture model to study neuron, astrocyte, and microglia interactions in neuroinflammation. J Neuroinflammation 17:155. 10.1186/s12974-020-01819-z 32393376 10.1186/s12974-020-01819-z PMC7216677

[CR43] Goshi N, Kim H, Girardi G et al (2023) Electrophysiological activity of primary cortical neuron-glia mixed cultures. Cells. 10.3390/cells12050821 36899957 10.3390/cells12050821 PMC10001406

[CR44] Grand RJ (2023) SARS-CoV-2 and the DNA damage response. J Gen Virol 104:001918. 10.1099/jgv.0.001918 37948194 10.1099/jgv.0.001918 PMC10768691

[CR45] Gupta T, Kumar M, Kaur UJ et al (2024) Mapping ACE2 and TMPRSS2 co-expression in human brain tissue: implications for SARS-CoV-2 neurological manifestations. J Neurovirol 30:316–326. 10.1007/s13365-024-01206-x 38600308 10.1007/s13365-024-01206-x

[CR46] Gusel’nikova VV, Korzhevskiy DE (2015) NeuN as a neuronal nuclear antigen and neuron differentiation marker. Acta Naturae 7:42–47 26085943 PMC4463411

[CR47] Harapan BN, Yoo HJ (2021) Neurological symptoms, manifestations, and complications associated with severe acute respiratory syndrome coronavirus 2 (SARS-CoV-2) and coronavirus disease 19 (COVID-19). J Neurol 268:3059–3071. 10.1007/s00415-021-10406-y 33486564 10.1007/s00415-021-10406-y PMC7826147

[CR48] Haverty R, McCormack J, Evans C et al (2024) SARS-CoV-2 infects neurons, astrocytes, choroid plexus epithelial cells and pericytes of the human central nervous system in vitro. J Gen Virol 105:1–11. 10.1099/jgv.0.002009 10.1099/jgv.0.002009 PMC11317966 38995681

[CR49] He N, Jin W-L, Lok K-H (2013) Amyloid-β(1–42) oligomer accelerates senescence in adult hippocampal neural stem/progenitor cells via formylpeptide receptor 2. Cell Death Dis 4:e924. 10.1038/cddis.2013.437 24263098 10.1038/cddis.2013.437 PMC3847315

[CR50] He M, Dong H, Huang Y et al (2016) Astrocyte-derived CCL2 is associated with M1 activation and recruitment of cultured microglial cells. Cell Physiol Biochem 38:859–870. 10.1159/000443040 26910882 10.1159/000443040

[CR51] Hernandez DG, Reed X, Singleton AB (2016) Genetics in Parkinson disease: Mendelian versus non-Mendelian inheritance. J Neurochem 139(Suppl):59–74. 10.1111/jnc.13593 27090875 10.1111/jnc.13593 PMC5155439

[CR52] Hoffmann M, Kleine-weber H, Schroeder S (2020) SARS-CoV-2 cell entry depends on ACE2 and TMPRSS2 and is blocked by a clinically proven protease inhibitor. Cell. 10.1016/j.cell.2020.02.052 32142651 10.1016/j.cell.2020.02.052 PMC7102627

[CR53] Howe CL, LaFrance-Corey RG, Goddery EN (2017) Neuronal CCL2 expression drives inflammatory monocyte infiltration into the brain during acute virus infection. J Neuroinflammation 14:238. 10.1186/s12974-017-1015-2 29202854 10.1186/s12974-017-1015-2 PMC5715496

[CR54] Hussmann KL, Fredericksen BL (2014) Differential induction of CCL5 by pathogenic and non-pathogenic strains of West Nile virus in brain endothelial cells and astrocytes. J Gen Virol 95:862–867. 10.1099/vir.0.060558-0 24413421 10.1099/vir.0.060558-0 PMC3973477

[CR55] Hwang M, Bergmann CC (2018) Alpha/Beta interferon (IFN-α/β) signaling in astrocytes mediates protection against viral encephalomyelitis and regulates IFN-γ-dependent responses. J Virol. 10.1128/JVI.01901-17 29491163 10.1128/JVI.01901-17 PMC5923078

[CR56] Israelow B, Song E, Mao T (2020) Mouse model of SARS-CoV-2 reveals inflammatory role of type i interferon signaling. J Exp Med. 10.1084/jem.20201241 32750141 10.1084/jem.20201241 PMC7401025

[CR57] Jiang H, Hyddmark EM V, Gordon S, et al (2022) Development of humanized ACE2 mouse and rat models for COVID‐19 research. FASEB J. 36

[CR58] Jeffries AM, Marriott I (2017) Human microglia and astrocytes express cGAS-STING viral sensing components. Neurosci Lett 658:53–56. 10.1016/j.neulet.2017.08.039 28830822 10.1016/j.neulet.2017.08.039 PMC5645252

[CR59] Jeong GU, Lyu J, Kim K-D et al (2022) SARS-CoV-2 infection of microglia elicits proinflammatory activation and apoptotic cell death. Microbiol Spectr 10:e0109122. 10.1128/spectrum.01091-22 35510852 10.1128/spectrum.01091-22 PMC9241873

[CR60] Jiao L, Yang Y, Yu W, et al (2020) SARS-CoV-2 Invades the Central Nervous System via the Olfactory Route in Rhesus Monkeys. SSRN Electron J 1–23. 10.2139/ssrn.3689615

[CR61] Joly-Amado A, Hunter J, Quadri Z et al (2020) CCL2 overexpression in the brain promotes glial activation and accelerates Tau pathology in a mouse model of tauopathy. Front Immunol 11:997. 10.3389/fimmu.2020.00997 32508844 10.3389/fimmu.2020.00997 PMC7251073

[CR62] Jorgačevski J, Potokar M (2023) Immune functions of astrocytes in viral neuroinfections. Int J Mol Sci. 10.3390/ijms24043514 36834929 10.3390/ijms24043514 PMC9960577

[CR63] Kanberg N, Simren J, Eden A et al (2021) Neurochemical signs of astrocytic and neuronal injury in acute COVID-19 normalizes during long-term follow-up. EBioMedicine. 10.1016/j.ebiom.2021.103512 34333238 10.1016/j.ebiom.2021.103512 PMC8320425

[CR64] Kato H, Takeuchi O, Mikamo-Satoh E et al (2008) Length-dependent recognition of double-stranded ribonucleic acids by retinoic acid–inducible gene-I and melanoma differentiation–associated gene 5. J Exp Med 205:1601–1610. 10.1084/jem.20080091 18591409 10.1084/jem.20080091 PMC2442638

[CR65] Käufer C, Schreiber CS, Hartke AS et al (2022) Microgliosis and neuronal proteinopathy in brain persist beyond viral clearance in SARS-CoV-2 hamster model. eBioMedicine 79:1–11. 10.1016/j.ebiom.2022.103999 10.1016/j.ebiom.2022.103999 PMC9013202 35439679

[CR66] Kircheis R, Haasbach E, Lueftenegger D et al (2020) NF-κB pathway as a potential target for treatment of critical stage COVID-19 patients. Front Immunol 11:598444. 10.3389/fimmu.2020.598444 33362782 10.3389/fimmu.2020.598444 PMC7759159

[CR67] Kobayashi K, Ichikawa M, Muramoto K et al (1993) Formation and maturation of synapses in primary cultures of rat cerebral cortical cells: an electron microscopic study. Neurosci Res 16:95–103. 10.1016/0168-0102(93)90076-3 8387174 10.1016/0168-0102(93)90076-3

[CR68] Kok JR, Palminha NM, Dos Santos Souza C et al (2021) DNA damage as a mechanism of neurodegeneration in ALS and a contributor to astrocyte toxicity. Cell Mol Life Sci 78:5707–5729. 10.1007/s00018-021-03872-0 34173837 10.1007/s00018-021-03872-0 PMC8316199

[CR69] Kong W, Montano M, Corley MJ et al (2022) Neuropilin-1 mediates SARS-CoV-2 infection of astrocytes in brain organoids, inducing inflammation leading to dysfunction and death of neurons. Mbio. 10.1128/mbio.02308-22 36314791 10.1128/mbio.02308-22 PMC9765283

[CR70] Krasemann S, Dittmayer C, von Stillfried S et al (2022) Assessing and improving the validity of COVID-19 autopsy studies - A multicentre approach to establish essential standards for immunohistochemical and ultrastructural analyses. eBioMedicine 83:1–16. 10.1016/j.ebiom.2022.104193 10.1016/j.ebiom.2022.104193 PMC9344879 35930888

[CR71] Lama L, Adura C, Xie W et al (2019) Development of human cGAS-specific small-molecule inhibitors for repression of dsDNA-triggered interferon expression. Nat Commun 10:1–14. 10.1038/s41467-019-08620-4 31113940 10.1038/s41467-019-08620-4 PMC6529454

[CR72] Lee S, Yu Y, Trimpert J et al (2021) Virus-induced senescence is a driver and therapeutic target in COVID-19. Nature 599:283–289. 10.1038/s41586-021-03995-1 34517409 10.1038/s41586-021-03995-1

[CR73] Licastro D, Rajasekharan Sreejith DMS, Segat L et al (2020) Isolation and Full-Length Genome Characterization of SARSCoV- 2 from COVID-19 Cases in Northern Italy. J Virol 94:10–128 10.1128/JVI.00543-20 PMC7269454 32238585

[CR74] López-Otín C, Blasco MA, Partridge L (2023) Hallmarks of aging: an expanding universe. Cell 186:243–278. 10.1016/j.cell.2022.11.001 36599349 10.1016/j.cell.2022.11.001

[CR75] Low RN, Low RJ, Akrami A (2023) A review of cytokine-based pathophysiology of long COVID symptoms. Front Med 10:1011936. 10.3389/fmed.2023.1011936 10.3389/fmed.2023.1011936 PMC10103649 37064029

[CR76] Luo Y, Berman MA, Zhai Q et al (2002) RANTES stimulates inflammatory cascades and receptor modulation in murine astrocytes. Glia 39:19–30. 10.1002/glia.10079 12112372 10.1002/glia.10079

[CR77] Ma W, Lu K, Liang H-M, Zhang J-Y (2023) Synapsin 1 ameliorates cognitive impairment and neuroinflammation in rats with Alzheimer’s disease: an experimental and bioinformatics study. Curr Alzheimer Res 20:648–659. 10.2174/0115672050276594231229050906 38213171 10.2174/0115672050276594231229050906

[CR78] Mahmud M, Pulizzi R, Vasilak E, Giugliano M (2014) Qspike tools: a generic framework for parallel batch preprocessing of extracellular neuronal signals recorded by substrate microelectrode arrays. Front Neuroinform 8:1–14. 10.3389/fninf.2014.00026 24678297 10.3389/fninf.2014.00026 PMC3958706

[CR79] Malik JR, Acharya A, Avedissian SN (2023) ACE-2, TMPRSS2, and neuropilin-1 receptor expression on human brain astrocytes and pericytes and SARS-CoV-2 infection kinetics. Int J Mol Sci. 10.3390/ijms24108622 37239978 10.3390/ijms24108622 PMC10218482

[CR80] Manzati M, Sorbo T, Giugliano M, Ballerini L (2020) Foetal neural progenitors contribute to postnatal circuits formation ex vivo: an electrophysiological investigation. Mol Brain 13:78. 10.1186/s13041-020-00619-z 32430072 10.1186/s13041-020-00619-z PMC7236481

[CR81] Mao L, Jin H, Wang M (2020) Neurologic manifestations of hospitalized patients with coronavirus disease 2019 in Wuhan, China. JAMA Neurol 77:683–690. 10.1001/jamaneurol.2020.1127 32275288 10.1001/jamaneurol.2020.1127 PMC7149362

[CR82] Marx S, Kümmerer BM, Grützner C et al (2022) RIG-I-induced innate antiviral immunity protects mice from lethal SARS-CoV-2 infection. Mol Ther Nucleic Acids 27:1225–1234. 10.1016/j.omtn.2022.02.008 35186439 10.1016/j.omtn.2022.02.008 PMC8841011

[CR83] Matelski L, Morgan RK, Grodzki AC et al (2021) Effects of cytokines on nuclear factor-kappa B, cell viability, and synaptic connectivity in a human neuronal cell line. Mol Psychiatry 26:875–887. 10.1038/s41380-020-0647-2 31965031 10.1038/s41380-020-0647-2 PMC7371517

[CR84] McMahon CL, Staples H, Gazi M et al (2021) SARS-CoV-2 targets glial cells in human cortical organoids. Stem Cell Reports 16:1156–1164. 10.1016/j.stemcr.2021.01.016 33979600 10.1016/j.stemcr.2021.01.016 PMC8111796

[CR85] Meinhardt J, Radke J, Dittmayer C et al (2021) Olfactory transmucosal SARS-CoV-2 invasion as a port of central nervous system entry in individuals with COVID-19. Nat Neurosci 24:168–175. 10.1038/s41593-020-00758-5 33257876 10.1038/s41593-020-00758-5

[CR86] Melo Dos Santos LS, Trombetta-Lima M, Eggen B, Demaria M (2024) Cellular senescence in brain aging and neurodegeneration. Ageing Res Rev 93:102141. 10.1016/j.arr.2023.102141 38030088 10.1016/j.arr.2023.102141

[CR87] Mesci P, de Souza JS, Martin-Sancho L et al (2022) SARS-CoV-2 infects human brain organoids causing cell death and loss of synapses that can be rescued by treatment with Sofosbuvir. PLoS Biol 20:1–23. 10.1371/journal.pbio.3001845 10.1371/journal.pbio.3001845 PMC9632769 36327326

[CR88] Miot EF, Worthington BM, Ng KH et al (2022) Surveillance of rodent pests for SARS-CoV-2 and other coronaviruses, Hong Kong. Emerg Infect Dis 28:467–470. 10.3201/eid2802.211586 35076003 10.3201/eid2802.211586 PMC8798707

[CR89] Mladinich MC, Conde JN, Schutt WR et al (2021) Blockade of autocrine ccl5 responses inhibits zika virus persistence and spread in human brain microvascular endothelial cells. Mbio 12:1–16. 10.1128/mBio.01962-21 10.1128/mBio.01962-21 PMC8406327 34399621

[CR90] Monje M, Iwasaki A (2022) The neurobiologyoflongCOVID. Neuron. 10.1016/j.neuron.2022.10.00636327895

[CR91] Moreau GB, Burgess SL, Sturek JM et al (2020) Evaluation of K18-hACE2 mice as a model of SARS-CoV-2 infection. Am J Trop Med Hyg 103:1215–1219. 10.4269/ajtmh.20-0762 32723427 10.4269/ajtmh.20-0762 PMC7470527

[CR92] Neufeldt CJ, Cerikan B, Cortese M et al (2022) SARS-CoV-2 infection induces a pro-inflammatory cytokine response through cGAS-STING and NF-κB. Commun Biol. 10.1038/s42003-021-02983-5 35022513 10.1038/s42003-021-02983-5 PMC8755718

[CR93] O’Mahoney LL, Routen A, Gillies C et al (2023) The prevalence and long-term health effects of Long Covid among hospitalised and non-hospitalised populations: A systematic review and meta-analysis. EClinicalMedicine 55:101762. 10.1016/j.eclinm.2022.101762 36474804 10.1016/j.eclinm.2022.101762 PMC9714474

[CR94] Padmashri R, Ren B, Oldham B et al (2021) Modeling human-specific interlaminar astrocytes in the mouse cerebral cortex. J Comp Neurol 529:802–810. 10.1002/cne.24979 32639590 10.1002/cne.24979 PMC7818222

[CR95] Parra B, Hinton DR, Marten NW et al (1999) IFN-gamma is required for viral clearance from central nervous system oligodendroglia. J Immunol 162:1641–1647 9973424

[CR96] Pellegrini L, Albecka A, Mallery DL et al (2020) Sars-cov-2 infects the brain choroid plexus and disrupts the blood-CSF barrier in human brain organoids. Cell Stem Cell 27:951-961.e5. 10.1016/j.stem.2020.10.001 33113348 10.1016/j.stem.2020.10.001 PMC7553118

[CR97] Perea G, Navarrete M, Araque A (2009) Tripartite synapses: astrocytes process and control synaptic information. Trends Neurosci 32:421–431. 10.1016/j.tins.2009.05.001 19615761 10.1016/j.tins.2009.05.001

[CR98] Pfefferkorn C, Kallfass C, Lienenklaus S et al (2016) Abortively infected astrocytes appear to represent the main source of interferon beta in the virus-infected brain. J Virol 90:2031–2038. 10.1128/JVI.02979-15 26656686 10.1128/JVI.02979-15 PMC4733997

[CR99] Piacentini R, Li Puma DD, Ripoli C (2015) Herpes simplex virus type-1 infection induces synaptic dysfunction in cultured cortical neurons via GSK-3 activation and intraneuronal amyloid-β protein accumulation. Sci Rep 5:15444. 10.1038/srep15444 26487282 10.1038/srep15444 PMC4614347

[CR100] Pozzi D, Meneghetti N, Roy A (2020) The role of network architecture in the onset of spontaneous activity”. Stemedicine. 10.37175/stemedicine.v1i1.1

[CR101] Proust A, Queval CJ, Harvey R et al (2023) Differential effects of SARS-CoV-2 variants on central nervous system cells and blood–brain barrier functions. J Neuroinflammation 20:1–17. 10.1186/s12974-023-02861-3 37537664 10.1186/s12974-023-02861-3 PMC10398935

[CR102] Puray-Chavez M, Eschbach JE, Xia M et al (2024) A basally active cGAS-STING pathway limits SARS-CoV-2 replication in a subset of ACE2 positive airway cell models. Nat Commun 15:8394. 10.1038/s41467-024-52803-7 39333139 10.1038/s41467-024-52803-7 PMC11437049

[CR103] Queiroz MAF, Brito WRDS, Pereira KAS (2024) Severe COVID-19 and long COVID are associated with high expression of STING, cGAS and IFN-α. Sci Rep 14:4974. 10.1038/s41598-024-55696-0 38424312 10.1038/s41598-024-55696-0 PMC10904751

[CR104] Qiao H, Qu Y, Qiu L, et al (2024) A Mouse-adapted SARS-CoV-2 Model for Investigating Post-acute Sequelae of COVID infection. bioRxiv 2024.11.10.622868. 10.1101/2024.11.10.622868

[CR105] Quiroga RQ, Nadasdy Z, Ben-Shaul Y (2004) Unsupervised spike detection and sorting with wavelets and superparamagnetic clustering. Neural Comput 16(8):1661–1687. 10.1162/089976604774201631 15228749 10.1162/089976604774201631

[CR106] Radke J, Meinhardt J, Aschman T et al (2024) Proteomic and transcriptomic profiling of brainstem, cerebellum and olfactory tissues in early- and late-phase COVID-19. Nat Neurosci 27:409–420. 10.1038/s41593-024-01573-y 38366144 10.1038/s41593-024-01573-y

[CR107] Rajasekharan S, Milan Bonotto R, Nascimento Alves L et al (2021) Inhibitors of protein glycosylation are active against the coronavirus severe acute respiratory syndrome coronavirus SARS-CoV-2. Viruses. 10.3390/v13050808 33946304 10.3390/v13050808 PMC8144969

[CR108] Ramesh G, MacLean AG, Philipp MT (2013) Cytokines and chemokines at the crossroads of neuroinflammation, neurodegeneration, and neuropathic pain. Mediators Inflamm 2013:480739. 10.1155/2013/480739 23997430 10.1155/2013/480739 PMC3753746

[CR109] Rathnasinghe R, Strohmeier S, Amanat F et al (2020) Comparison of transgenic and adenovirus hACE2 mouse models for SARS-CoV-2 infection. Emerg Microbes Infect 9:2433–2445. 10.1080/22221751.2020.1838955 33073694 10.1080/22221751.2020.1838955 PMC7655046

[CR110] Rho MB, Wesselingh S, Glass JD et al (1995) A potential role for interferon-alpha in the pathogenesis of HIV-associated dementia. Brain Behav Immun 9:366–377. 10.1006/brbi.1995.1034 8903853 10.1006/brbi.1995.1034

[CR111] Schindelin J, Arganda-Carreras I, Frise E et al (2012) Fiji: an open-source platform for biological-image analysis. Nat Methods 9:676–682. 10.1038/nmeth.2019 22743772 10.1038/nmeth.2019 PMC3855844

[CR112] Shabab T, Khanabdali R, Moghadamtousi SZ et al (2017) Neuroinflammation pathways: a general review. Int J Neurosci 127:624–633. 10.1080/00207454.2016.1212854 27412492 10.1080/00207454.2016.1212854

[CR113] Shu C, Li X, Li P (2014) The mechanism of double-stranded DNA sensing through the cGAS-STING pathway. Cytokine Growth Factor Rev 25:641–648. 10.1016/j.cytogfr.2014.06.006 25007740 10.1016/j.cytogfr.2014.06.006 PMC4254336

[CR114] Sliter DA, Martinez J, Hao L et al (2018) Parkin and PINK1 mitigate STING-induced inflammation. Nature 561:258–262. 10.1038/s41586-018-0448-9 30135585 10.1038/s41586-018-0448-9 PMC7362342

[CR115] Smale ST (2010) Selective transcription in response to an inflammatory stimulus. Cell 140:833–844. 10.1016/j.cell.2010.01.037 20303874 10.1016/j.cell.2010.01.037 PMC2847629

[CR116] Song E, Zhang C, Israelow B et al (2021) Neuroinvasion of SARS-CoV-2 in human and mouse brain. J Exp Med 218:20202135 10.1084/jem.20202135 PMC7808299 33433624

[CR117] Stadlbauer D, Amanat F, Chromikova V et al (2020) SARS-CoV-2 Seroconversion in Humans: A Detailed Protocol for a Serological Assay, Antigen Production, and Test Setup. Curr Protoc Microbiol 57:1–15. 10.1002/cpmc.100 10.1002/cpmc.100 PMC7235504 32302069

[CR118] Stein SR, Ramelli SC, Grazioli A et al (2022) SARS-CoV-2 infection and persistence in the human body and brain at autopsy. Nature 612:758–763. 10.1038/s41586-022-05542-y 36517603 10.1038/s41586-022-05542-y PMC9749650

[CR119] Sun X, Wang T, Cai D et al (2020) Cytokine and Growth Factor Reviews Cytokine storm intervention in the early stages of COVID-19 pneumonia. Cytokine Growth Factor Rev. 53:38–42 32360420 10.1016/j.cytogfr.2020.04.002 PMC7182527

[CR120] Talbot EJ, Joshi L, Thornton P et al (2024) cGAS-STING signalling regulates microglial chemotaxis in genome instability. Nucleic Acids Res 52:1188–1206. 10.1093/nar/gkad1184 38084916 10.1093/nar/gkad1184 PMC10853792

[CR121] Tan P-H, Ji J, Hsing C-H (2022) Emerging roles of type-I interferons in neuroinflammation, neurological diseases, and long-haul COVID. Int J Mol Sci. 10.3390/ijms232214394 36430870 10.3390/ijms232214394 PMC9696119

[CR122] Thorne LG, Reuschl A, Zuliani-Alvarez L et al (2021) SARS-CoV-2 sensing by RIG-I and MDA5 links epithelial infection to macrophage inflammation. EMBO J 40:e107826. 10.15252/embj.2021107826 34101213 10.15252/embj.2021107826 PMC8209947

[CR123] Tremblay MÈ, Stevens B, Sierra A et al (2011) The role of microglia in the healthy brain. J Neurosci 31(45):16064–16069. 10.1523/JNEUROSCI.4158-11.2011 22072657 10.1523/JNEUROSCI.4158-11.2011 PMC6633221

[CR124] Ubogu EE, Callahan MK, Tucky BH, Ransohoff RM (2006) Determinants of CCL5-driven mononuclear cell migration across the blood-brain barrier. Implications for therapeutically modulating neuroinflammation. J Neuroimmunol 179:132–144. 10.1016/j.jneuroim.2006.06.004 16857269 10.1016/j.jneuroim.2006.06.004

[CR125] Usai C, Mateu L, Brander C et al (2023) Animal models to study the neurological manifestations of the post-COVID-19 condition. Lab Anim 52:202–210. 10.1038/s41684-023-01231-z 10.1038/s41684-023-01231-z PMC10462483 37620562

[CR126] Victor J, Deutsch J, Whitaker A et al (2021) SARS-CoV-2 triggers DNA damage response in Vero E6 cells. Biochem Biophys Res Commun 579:141–145. 10.1016/j.bbrc.2021.09.024 34600299 10.1016/j.bbrc.2021.09.024 PMC8440005

[CR127] Vieira SM, Lemos HP, Grespan R et al (2009) A crucial role for TNF‐α in mediating neutrophil influx induced by endogenously generated or exogenous chemokines, KC/CXCL1 and LIX/CXCL5. Br J Pharmacol 158:779–789. 10.1111/j.1476-5381.2009.00367.x 19702783 10.1111/j.1476-5381.2009.00367.x PMC2765597

[CR128] Vincent J, Adura C, Gao P (2017) Small molecule inhibition of cGAS reduces interferon expression in primary macrophages from autoimmune mice. Nat Commun 8:750. 10.1038/s41467-017-00833-9 28963528 10.1038/s41467-017-00833-9 PMC5622107

[CR129] Virhammar J, Kumlien E, Fällmar D et al (2020) Acute necrotizing encephalopathy with SARS-CoV-2 RNA confirmed in cerebrospinal fluid. Neurology 95:445–449. 10.1212/WNL.0000000000010250 32586897 10.1212/WNL.0000000000010250 PMC7538220

[CR130] Wagenaar DA, Pine J, Potter SM (2004) Effective parameters for stimulation of dissociated cultures using multi-electrode arrays. J Neurosci Methods 138:27–37. 10.1016/j.jneumeth.2004.03.005 15325108 10.1016/j.jneumeth.2004.03.005

[CR131] Wake H, Moorhouse AJ, Jinno S (2009) Resting microglia directly monitor the functional state of synapses in vivo and determine the fate of ischemic terminals. J Neurosci 29:3974–3980. 10.1523/JNEUROSCI.4363-08.2009 19339593 10.1523/JNEUROSCI.4363-08.2009 PMC6665392

[CR132] Wälchli T, Ghobrial M, Schwab M (2024) Single-cell atlas of the human brain vasculature across development, adulthood and disease. Nature 632:603–613. 10.1038/s41586-024-07493-y 38987604 10.1038/s41586-024-07493-y PMC11324530

[CR133] Wang Y, Lenoch J, Kohler D et al (2023) SARS-CoV-2 Exposure in Norway Rats (Rattus norvegicus) from New York City. Mbio 14:e0362122. 10.1128/mbio.03621-22 36892291 10.1128/mbio.03621-22 PMC10127689

[CR134] Werneburg S, Feinberg PA, Johnson KM, Schafer DP (2017) A microglia-cytokine axis to modulate synaptic connectivity and function. Curr Opin Neurobiol 47:138–145. 10.1016/j.conb.2017.10.002 29096242 10.1016/j.conb.2017.10.002 PMC5797987

[CR135] Winkler ES, Chen RE, Alam F et al (2022) SARS-CoV-2 Causes Lung Infection without Severe Disease in Human ACE2 Knock-In Mice. J Virol 96:e0151121. 10.1128/JVI.01511-21 34668780 10.1128/JVI.01511-21 PMC8754206

[CR136] Xia T, Yi X-M, Wu X et al (2019) PTPN1/2-mediated dephosphorylation of MITA/STING promotes its 20S proteasomal degradation and attenuates innate antiviral response. Proc Natl Acad Sci U S A 116:20063–20069. 10.1073/pnas.1906431116 31527250 10.1073/pnas.1906431116 PMC6778251

[CR137] Yang Q-Q, Zhou J-W (2019) Neuroinflammation in the central nervous system: symphony of glial cells. Glia 67:1017–1035. 10.1002/glia.23571 30548343 10.1002/glia.23571

[CR138] Zevini A, Olagnier D, Hiscott J (2017) Crosstalk between cytoplasmic RIG-I and STING sensing pathways. Trends Immunol 38:194–205. 10.1016/j.it.2016.12.004 28073693 10.1016/j.it.2016.12.004 PMC5329138

[CR139] Zhang C, Cui H, Li E et al (2022) The SARS-CoV-2 B.1.351 variant can transmit in rats but not in mice. Front Immunol 13:869809. 10.3389/fimmu.2022.869809 35572504 10.3389/fimmu.2022.869809 PMC9095975

[CR140] Zhou B, Thao TTN, Hoffmann D (2021) SARS-CoV-2 spike D614G change enhances replication and transmission. Nature 592:122–127. 10.1038/s41586-021-03361-1 33636719 10.1038/s41586-021-03361-1

[CR141] Zhu N, Zhang D, Wang W et al (2020) A novel coronavirus from patients with pneumonia in China, 2019. N Engl J Med 382:727–733. 10.1056/nejmoa2001017 31978945 10.1056/NEJMoa2001017 PMC7092803

