# Supplementary figures and images for: SARS-CoV-2-induced damage to rat cortical neuronal networks ex vivo is mediated by the pro-inflammatory activation of the cGAS-STING pathway
Source: J Neurovirol. 2025 Oct 25;31(6):528–54. doi: 10.1007/s13365-025-01283-6 (PMC12701001; doi:10.1007/s13365-025-01283-6)

A

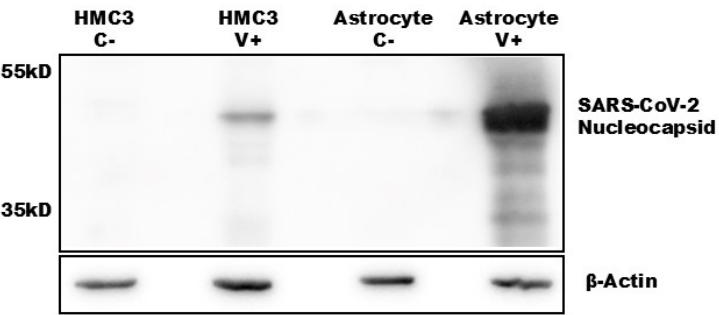

B

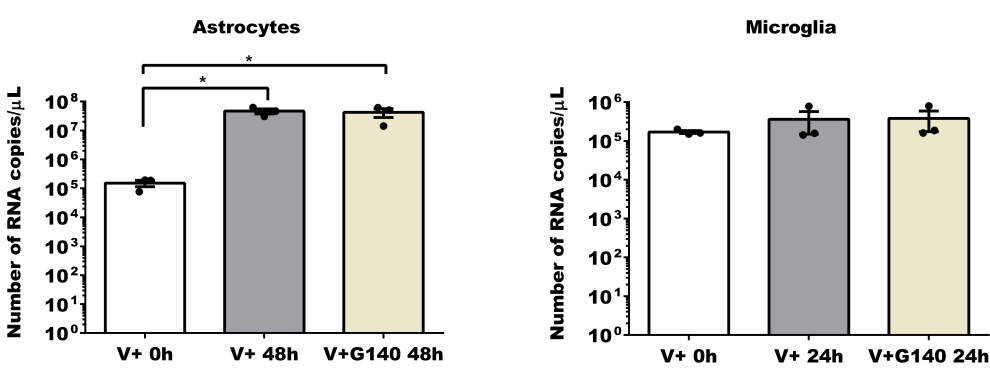

C

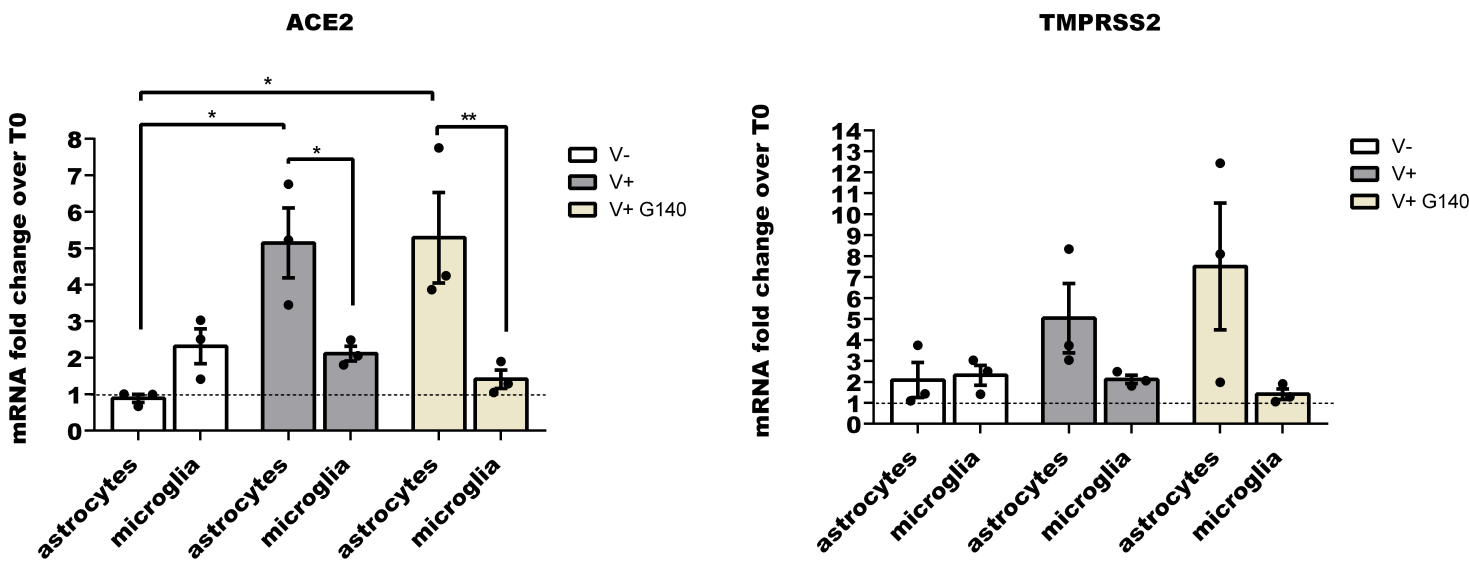

D

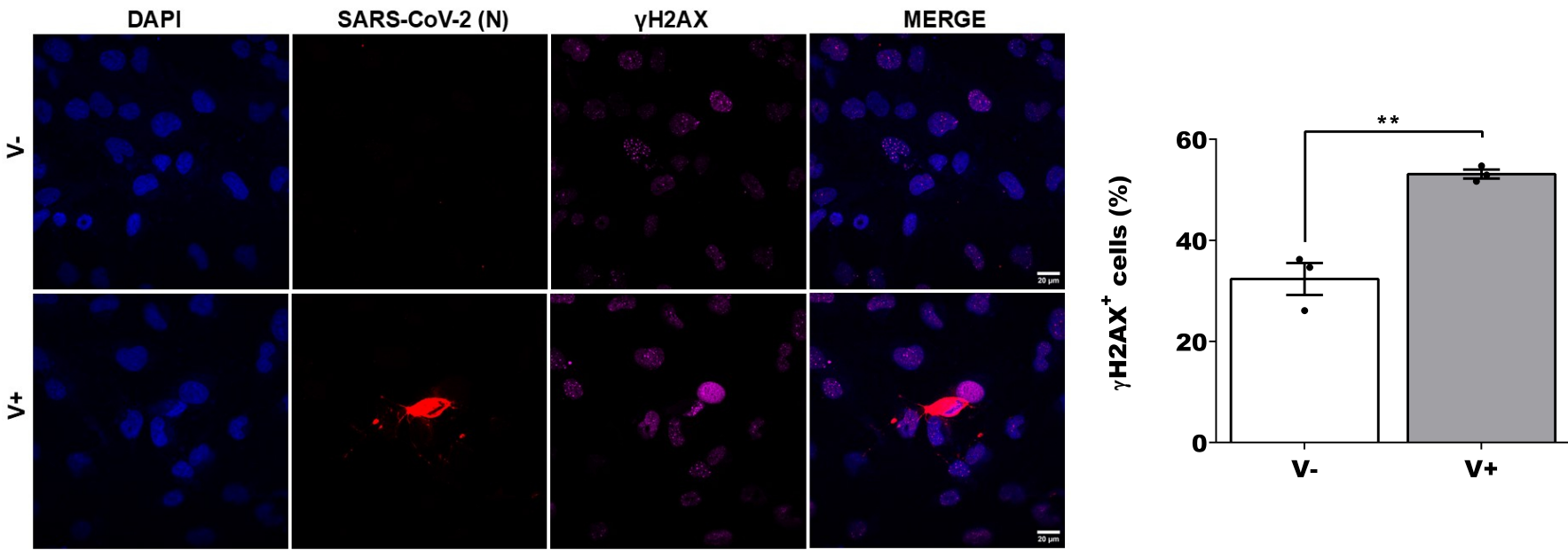

E

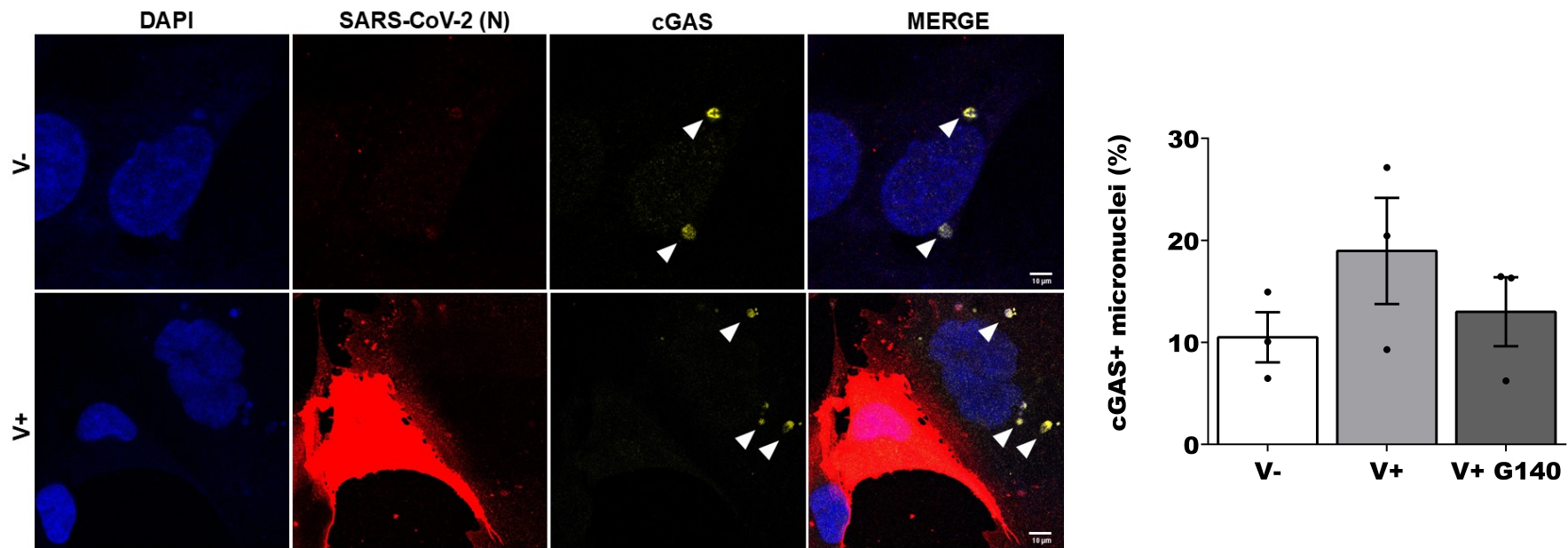

Supplement: Supplementary file 1 — Supplementary file1 (PDF 3045 KB) [file 13365_2025_1283_MOESM1_ESM.pdf]

A

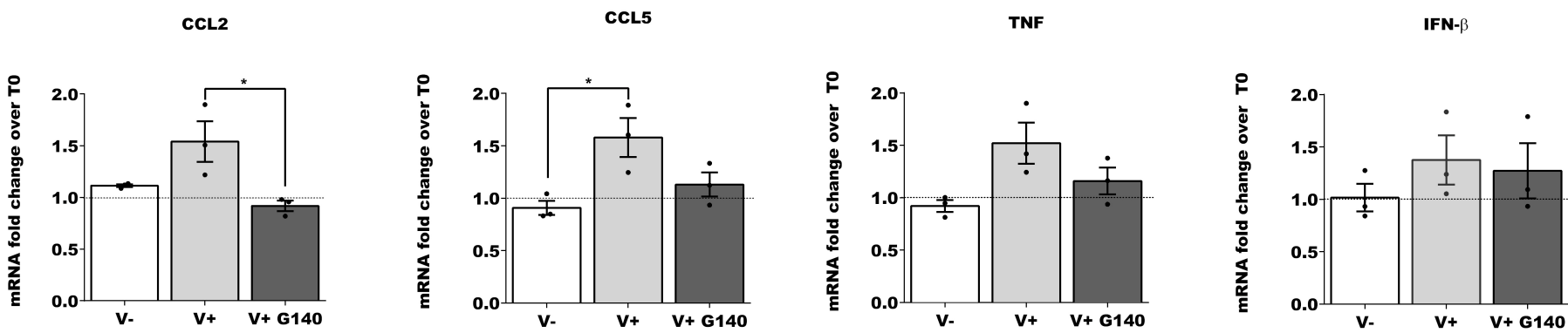

B

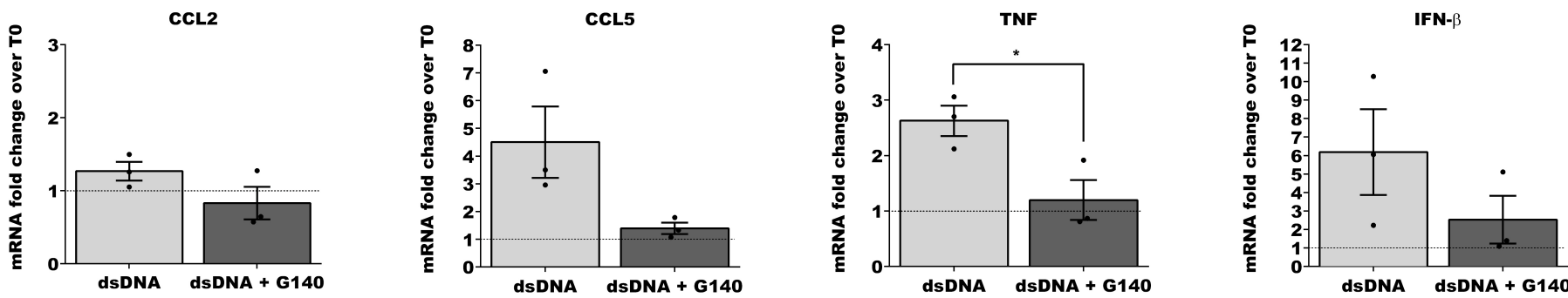

C

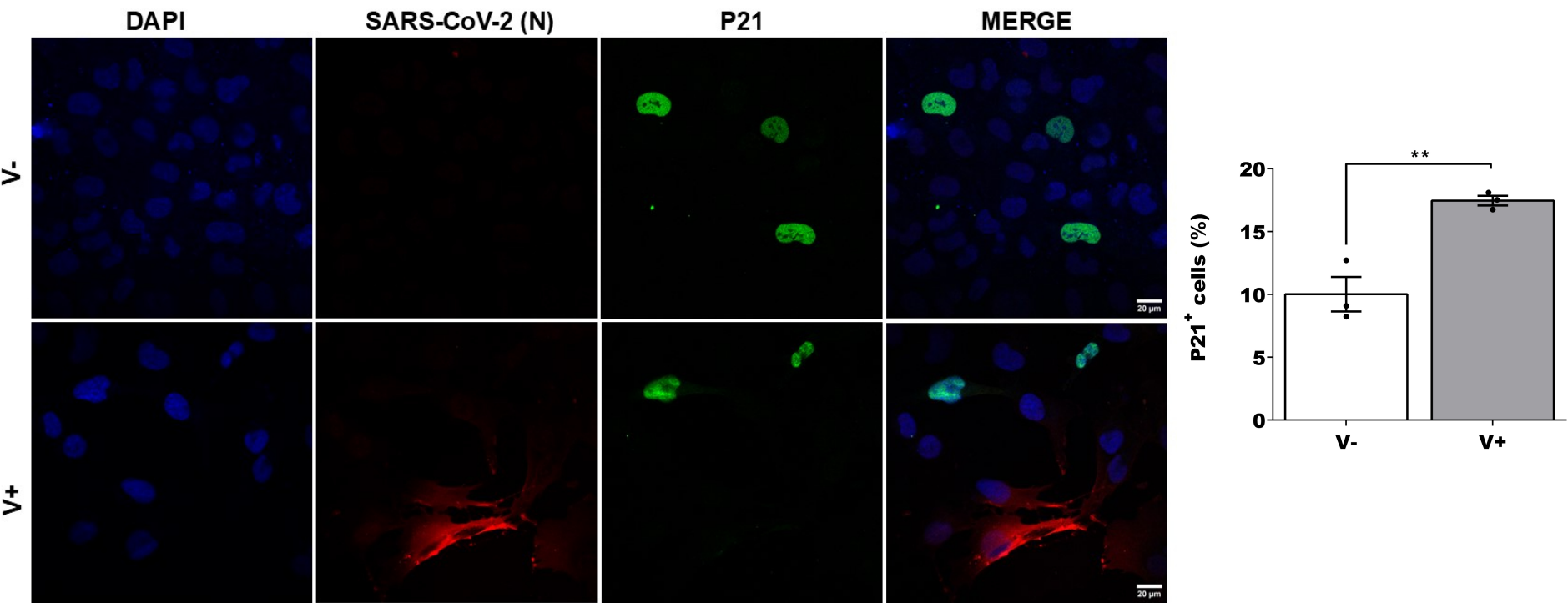

Supplement: Supplementary file 2 — Supplementary file2 (PDF 1371 KB) [file 13365_2025_1283_MOESM2_ESM.pdf]

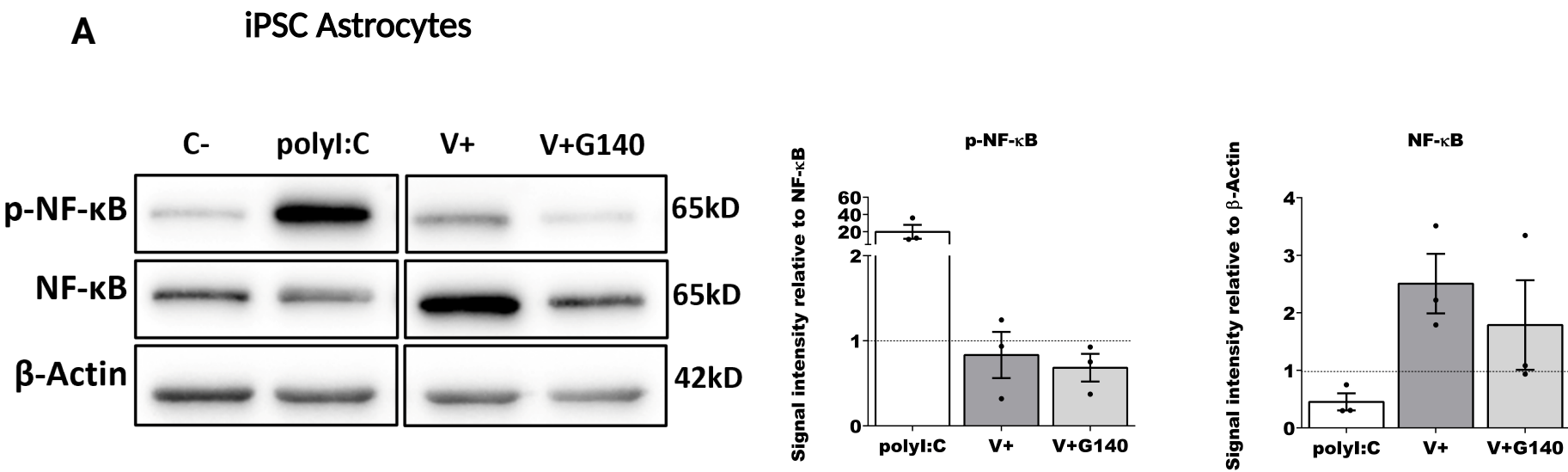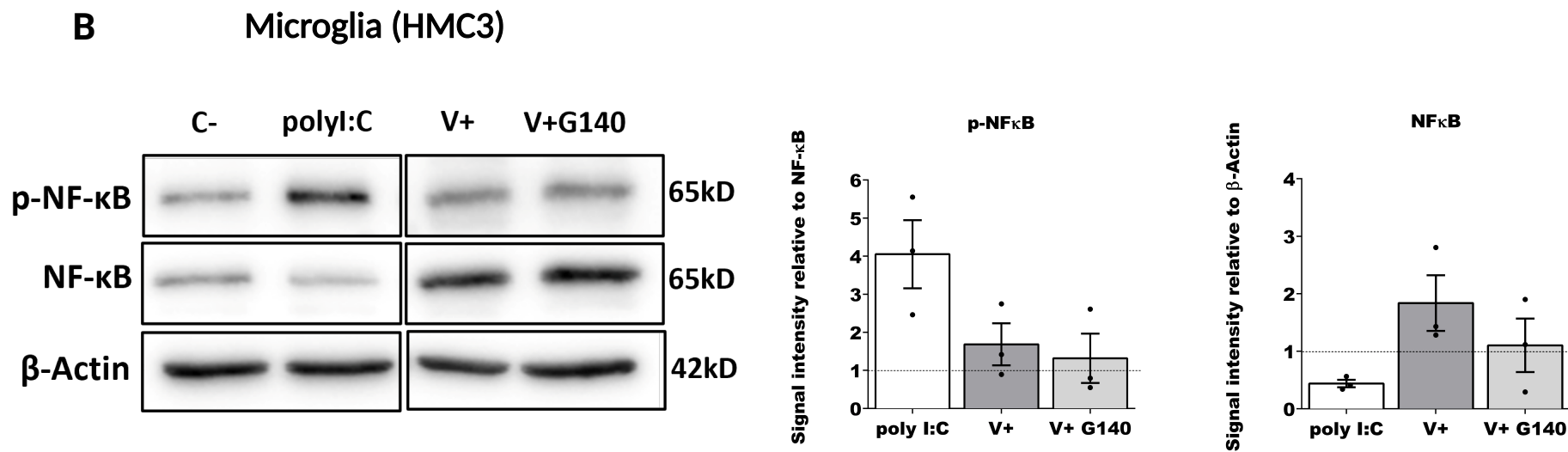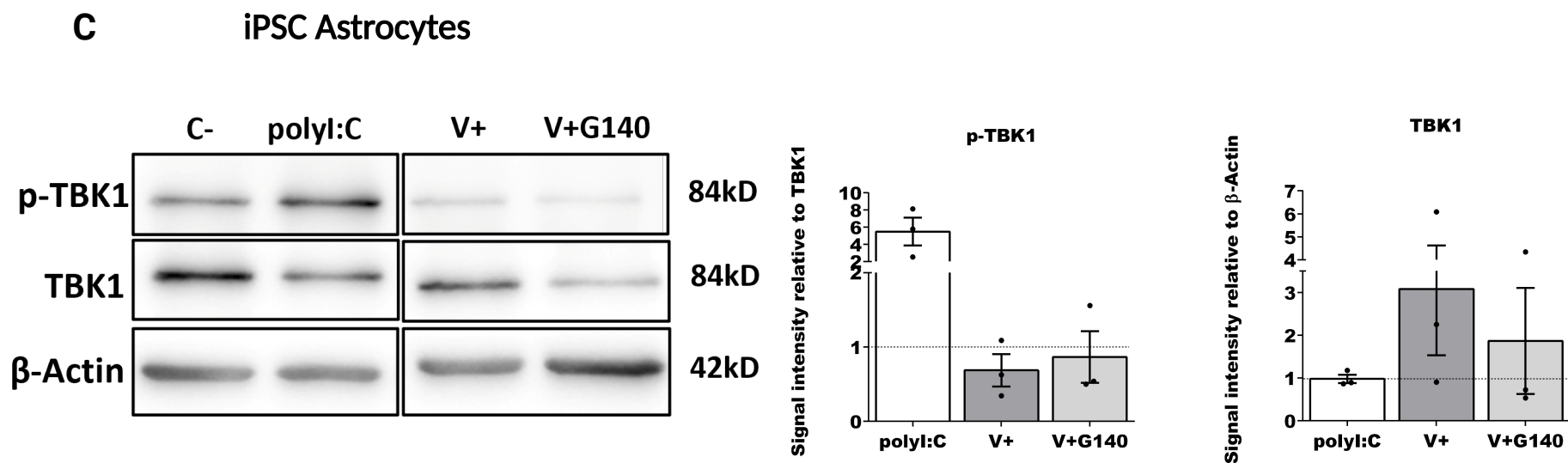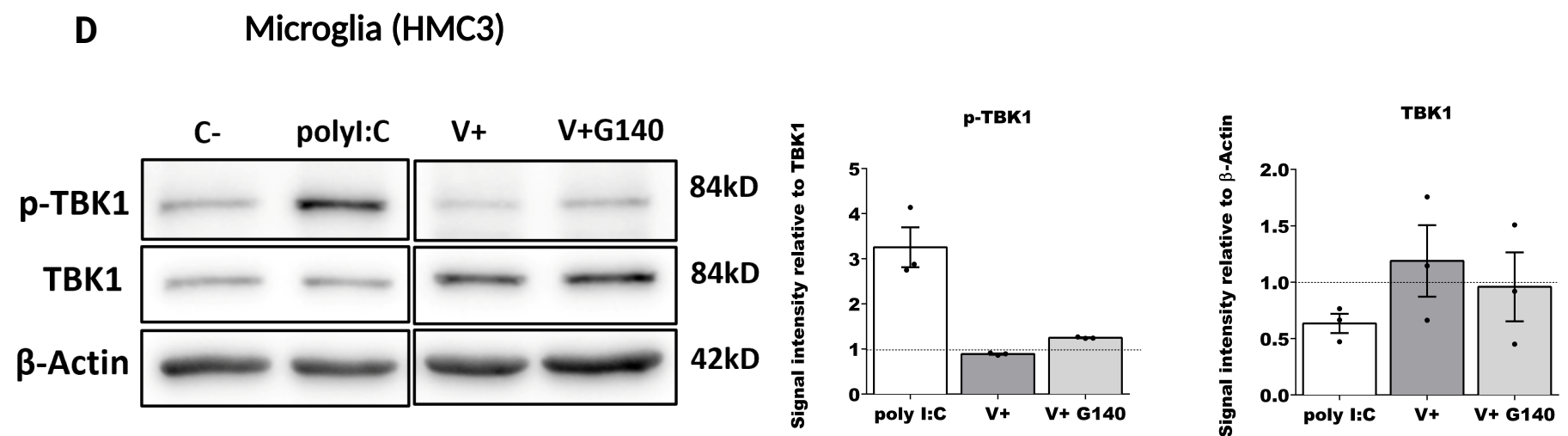

Supplement: Supplementary file 3 — Supplementary file3 (PDF 1886 KB) [file 13365_2025_1283_MOESM3_ESM.pdf]

A

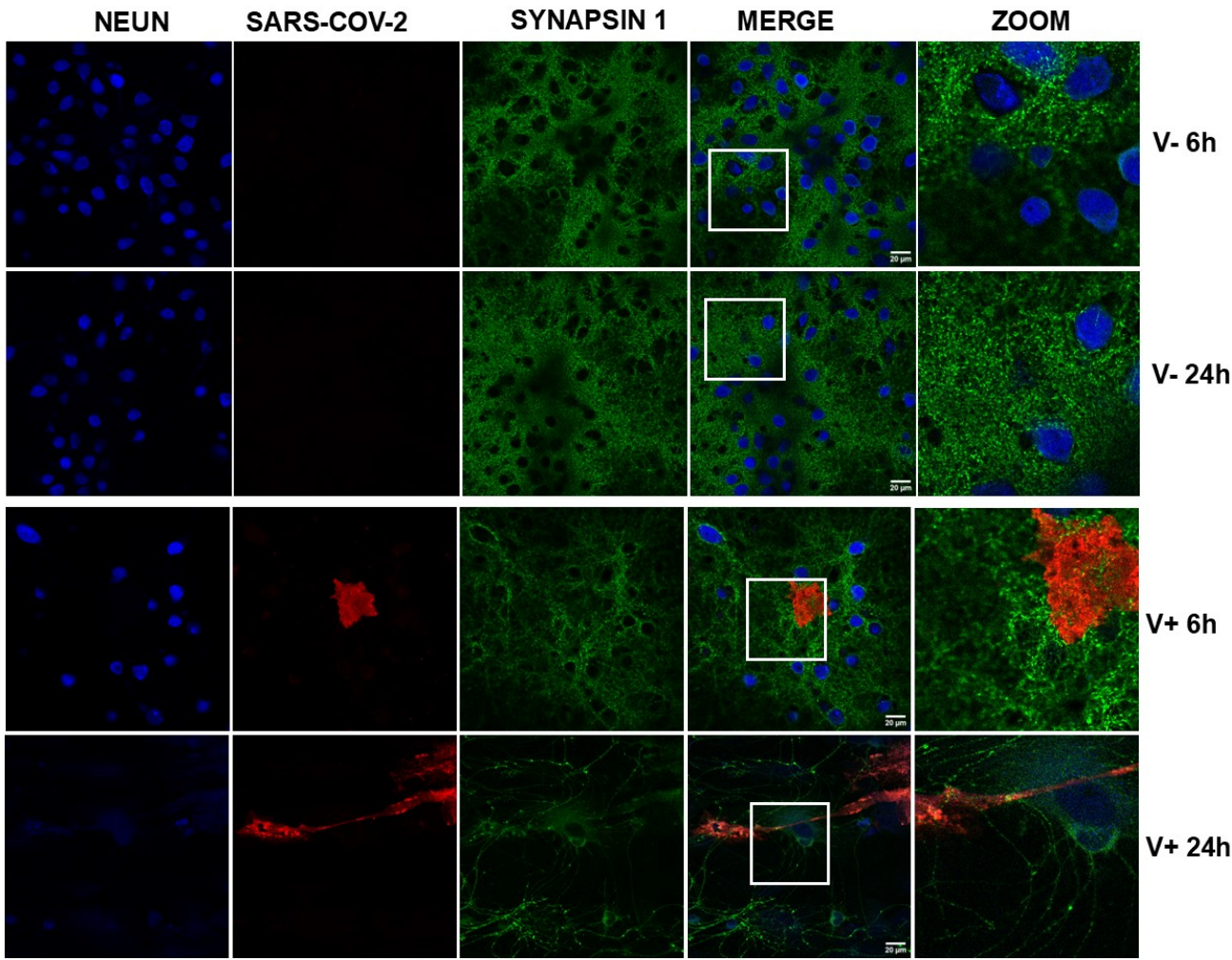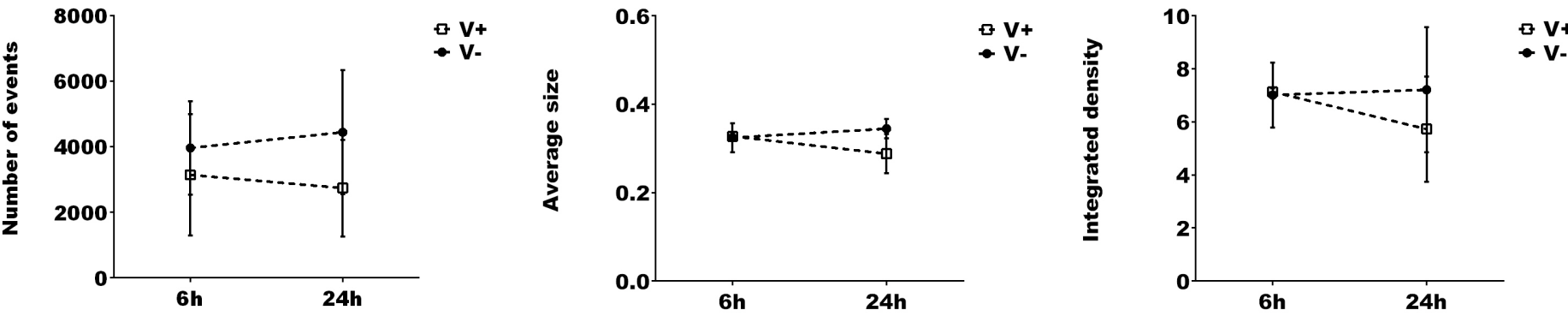

B

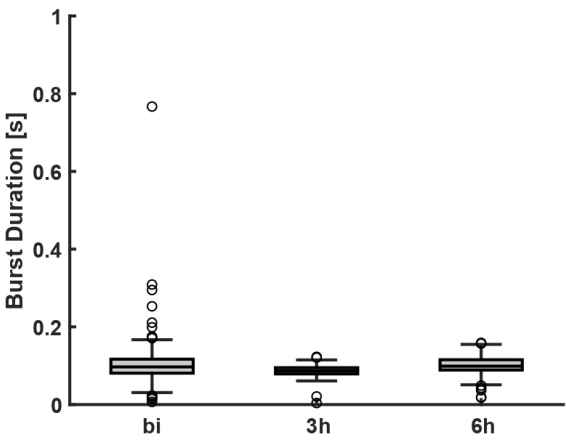

C

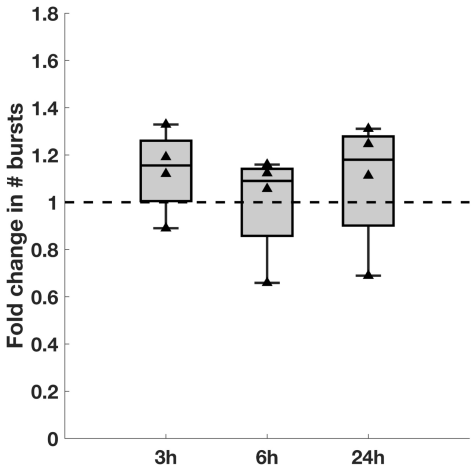

D

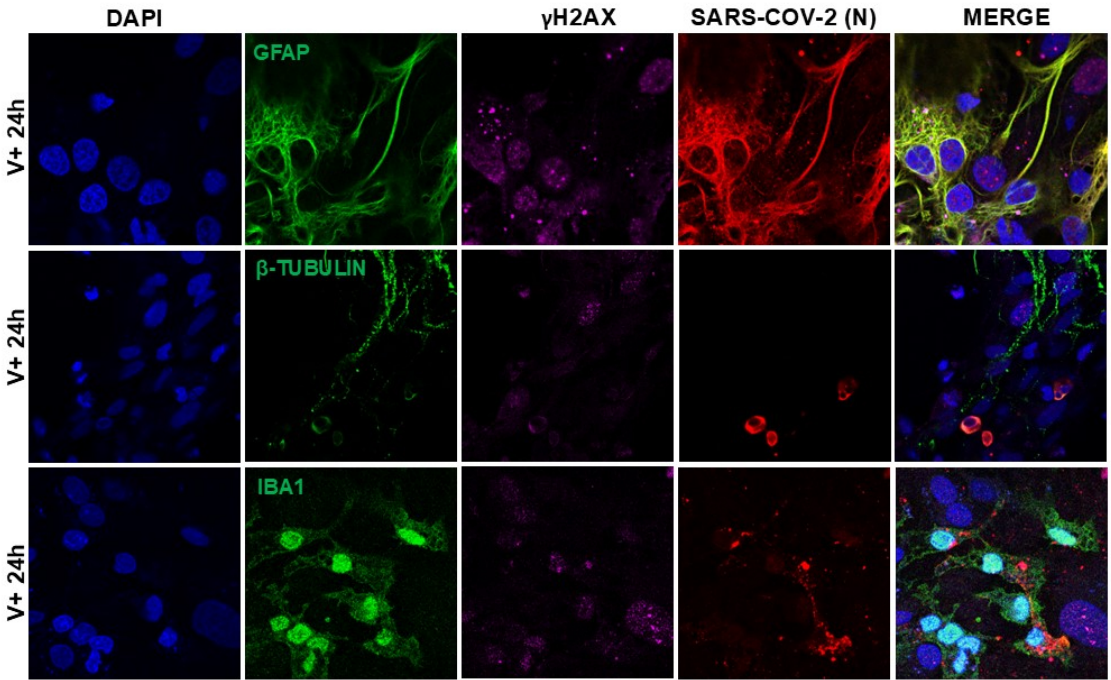

Supplement: Supplementary file 4 — Supplementary file4 (PDF 2440 KB) [file 13365_2025_1283_MOESM4_ESM.pdf]
