# Supplementary material for: SARS-CoV-2-induced damage to rat cortical neuronal networks ex vivo is mediated by the pro-inflammatory activation of the cGAS-STING pathway
Source: J Neurovirol. 2025 Oct 25;31(6):528–54. doi: 10.1007/s13365-025-01283-6 (PMC12701001; doi:10.1007/s13365-025-01283-6)
Supplement: Supplementary file 5 — Supplementary file5 (PDF 216 KB) [file 13365_2025_1283_MOESM5_ESM.pdf]

Figure number	Condition	Type of test (Normality)	P value	Type of test (variance)	P value
1C γH2AX	V-	Shapiro-Wilk	0,954056	Levene’s	0,287256
	V+	Shapiro-Wilk	0,831972	Levene’s	0,287256
1C cGAS	V-	Shapiro-Wilk	0,281483	Levene’s	0,281483
	V+	Shapiro-Wilk	0,892535	Levene’s	0,281483
	V+G140	Shapiro-Wilk	0,65199	Levene’s	0,281483
2A CCL2	V-	Shapiro-Wilk	0,445982	Levene’s	0,627356
	V+	Shapiro-Wilk	0,445982	Levene’s	0,627356
	V+G140	Shapiro-Wilk	0,897686	Levene’s	0,627356
2A CCL5	V-	Shapiro-Wilk	0,006422	Levene’s	0,871037
	V+	Shapiro-Wilk	0,386907	Levene’s	0,871037
	V+G140	Shapiro-Wilk	0,18768	Levene’s	0,871037
2A TNF	V-	Shapiro-Wilk	0,491128	Levene’s	0,52878
	V+	Shapiro-Wilk	0,450401	Levene’s	0,52878
	V+G140	Shapiro-Wilk	0,450401	Levene’s	0,52878
2A IFNβ	V-	Shapiro-Wilk	0,152162	Levene’s	0,143423
	V+	Shapiro-Wilk	0,85828	Levene’s	0,143423
	V+G140	Shapiro-Wilk	0,710835	Levene’s	0,143423
2B CCL2	dsDNA	Shapiro-Wilk	0,459331	Levene’s	0,702736
	dsDNA +G140	Shapiro-Wilk	0,459331	Levene’s	0,702736
2B CCL5	dsDNA	Shapiro-Wilk	0,001675	Levene’s	0,49717
	dsDNA +G140	Shapiro-Wilk	0,820725	Levene’s	0,49717
2B TNF	dsDNA	Shapiro-Wilk	0,49717	Levene’s	0,365405
	dsDNA +G140	Shapiro-Wilk	0,287504	Levene’s	0,365405
2B IFNβ	dsDNA	Shapiro-Wilk	0,365405	Levene’s	0,431445
	dsDNA +G140	Shapiro-Wilk	0,00981	Levene’s	0,431445
2C P21	V-	Shapiro-Wilk	0,899854	Levene’s	0,308683
	V+	Shapiro-Wilk	0,971736	Levene’s	0,308683
3A PFU/mL	3hpi	Shapiro-Wilk	0,049094	Levene’s	0,83181
	6hpi	Shapiro-Wilk	0,073643	Levene’s	0,83181
	24hpi	Shapiro-Wilk	0,099008	Levene’s	0,83181
3A PCR/supernatant	3hpi	Shapiro-Wilk	0,206137	Levene’s	0,685205
	6hpi	Shapiro-Wilk	0,219648	Levene’s	0,685205
	24hpi	Shapiro-Wilk	0,670636	Levene’s	0,685205
3A PCR/cell lysates	6hpi	Shapiro-Wilk	0,962021	Levene’s	0,462337
	24hpi	Shapiro-Wilk	0,31617	Levene’s	0,462337
3D β-tubulin III	3hpi	Shapiro-Wilk	0,138979	Levene’s	0,63232
	6hpi	Shapiro-Wilk	0,043401	Levene’s	0,63232
	24hpi	Shapiro-Wilk	0,726633	Levene’s	0,63232
3D GFAP	3hpi	Shapiro-Wilk	0,755873	Levene’s	0,540764
	6hpi	Shapiro-Wilk	0,755873	Levene’s	0,540764
	24hpi	Shapiro-Wilk	0,151821	Levene’s	0,540764
4B Inter-Burst Intervals	Bi	Anderson-Darling	< 0.00005	Levene’s	1.27 x 10^-4
	3h	Anderson-Darling	< 0.00005	Levene’s	1.27 x 10^-4

	6h	Anderson-Darling	< 0.00005	Levene's	1.27 x 10^-4
4C Burst Duration	Bi	Anderson-Darling	< 0.00005	Levene's	1.64 x 10^-22
	3h	Anderson-Darling	< 0.00005	Levene's	1.64 x 10^-22
	6h	Anderson-Darling	< 0.00005	Levene's	1.64 x 10^-22
5A yH2AX	V-	Shapiro-Wilk	0,532789	Levene's	0,910366
	UV	Shapiro-Wilk	0,277025	Levene's	0,910366
	V+	Shapiro-Wilk	0,191125	Levene's	0,910366
5B CCL2	V-	Shapiro-Wilk	0,163864	Levene's	0,627356
	UV	Shapiro-Wilk	0,577264	Levene's	0,627356
	V+	Shapiro-Wilk	0,238657	Levene's	0,627356
	V+ RU.521	Shapiro-Wilk	0,164566	Levene's	0,627356
5B CCL5	V-	Shapiro-Wilk	0,005269	Levene's	0,552404
	UV	Shapiro-Wilk	0,000449	Levene's	0,552404
	V+	Shapiro-Wilk	0,002013	Levene's	0,552404
	V+ RU.521	Shapiro-Wilk	0,001764	Levene's	0,552404
5B TNF	V-	Shapiro-Wilk	0,064657	Levene's	0,689561
	UV	Shapiro-Wilk	0,207675	Levene's	0,689561
	V+	Shapiro-Wilk	0,023804	Levene's	0,689561
	V+ RU.521	Shapiro-Wilk	0,135641	Levene's	0,689561
5B IFNβ	V-	Shapiro-Wilk	0,0715	Levene's	0,794206
	UV	Shapiro-Wilk	0,207327	Levene's	0,794206
	V+	Shapiro-Wilk	0,201853	Levene's	0,794206
	V+ RU.521	Shapiro-Wilk	0,000535	Levene's	0,794206
5C CCL2	dsDNA	Shapiro-Wilk	0,818252	Levene's	0,24043
	dsDNA +G140	Shapiro-Wilk	0,04379	Levene's	0,24043
5C CCL5	dsDNA	Shapiro-Wilk	0,082806	Levene's	0,354918
	dsDNA +G140	Shapiro-Wilk	0,093213	Levene's	0,354918
5C TNF	dsDNA	Shapiro-Wilk	0,288504	Levene's	0,313715
	dsDNA +G140	Shapiro-Wilk	0,705828	Levene's	0,313715
5C IFNβ	dsDNA	Shapiro-Wilk	0,715278	Levene's	0,527392
	dsDNA +G140	Shapiro-Wilk	0,581624	Levene's	0,527392
5D CCL2	V+6h	Shapiro-Wilk	0,089338	Levene's	0,47819
	V+24h	Shapiro-Wilk	0,089338	Levene's	0,47819
	UV 6h	ND	ND	ND	ND
	UV 24h	ND	ND	ND	ND
5D CCL5	V+6h	Shapiro-Wilk	0,636886	Levene's	0,461172
	V+24h	Shapiro-Wilk	0,522953	Levene's	0,461172
	UV 6h	ND	ND	ND	ND
	UV 24h	ND	ND	ND	ND
5D TNF	V+6h	Shapiro-Wilk	0,240763	Levene's	0,597105
	V+24h	Shapiro-Wilk	0,602433	Levene's	0,597105
	UV 6h	Shapiro-Wilk	ND	Levene's	0,597105
	UV 24h	Shapiro-Wilk	ND	Levene's	0,597105
5D IFNβ	V+6h	Shapiro-Wilk	0,046581	Levene's	0,83099

	V+24h	Shapiro-Wilk	0,830801	Levene's	0,83099
	UV 6h	ND	ND	ND	ND
	UV 24h	ND	ND	ND	ND
6A Number of bursts	Bi	Shapiro-Wilk	0.5671	Levene's	0.0038
	3h	Shapiro-Wilk	0.3453	Levene's	0.0038
	6h	Shapiro-Wilk	0.1684	Levene's	0.0038
	24h	Shapiro-Wilk	0.2018	Levene's	0.0038
6B Number of burst	Bi	Shapiro-Wilk	0.0213	Levene's	0.0333
	3h	Shapiro-Wilk	0.9046	Levene's	0.0333
	6h	Shapiro-Wilk	0.3251	Levene's	0.0333
Suppl 1B Astrocytes	V+ 0h	Shapiro-Wilk	0.058271	Levene's	0.324834
	V+ 48h	Shapiro-Wilk	0.954786	Levene's	0.324834
	V+G140 48h	Shapiro-Wilk	0.439727	Levene's	0.324834
Suppl 1B Microglia	V+ 0h	Shapiro-Wilk	0.379569	Levene's	0.645282
	V+ 24h	Shapiro-Wilk	0.039343	Levene's	0.645282
	V+G140 24h	Shapiro-Wilk	0.072168	Levene's	0.645282
Suppl 1C Astrocytes ACE2	V-	Shapiro-Wilk	0.071156	Levene's	0.501152
	V+	Shapiro-Wilk	0.916008	Levene's	0.501152
	V+G140	Shapiro-Wilk	0.169331	Levene's	0.501152
Suppl 1C Microglia ACE2	V-	Shapiro-Wilk	0.614869	Levene's	0.596802
	V+	Shapiro-Wilk	0.713756	Levene's	0.596802
	V+G140	Shapiro-Wilk	0.519927	Levene's	0.596802
Suppl 1C Astrocytes TMPRSS2	V-	Shapiro-Wilk	0.223624	Levene's	0.457585
	V+	Shapiro-Wilk	0.230365	Levene's	0.457585
	V+G140	Shapiro-Wilk	0.812026	Levene's	0.457585
Suppl 1C Microglia TMPRSS2	V-	Shapiro-Wilk	0.787161	Levene's	0.93499
	V+	Shapiro-Wilk	0.650726	Levene's	0.93499
	V+G140	Shapiro-Wilk	0.302853	Levene's	0.93499
Suppl 1D γH2AX	V-	Shapiro-Wilk	0,277527	Levene's	0,427559
	V+	Shapiro-Wilk	0,797638	Levene's	0,427559
Suppl 1E cGAS	V-	Shapiro-Wilk	0,833981	Levene's	0,720041
	V+	Shapiro-Wilk	0,726226	Levene's	0,720041
	V+G140	Shapiro-Wilk	0,021172	Levene's	0,720041
Suppl 2A CCL2	V-	Shapiro-Wilk	0,577752	Levene's	0,168595
	V+	Shapiro-Wilk	0,832746	Levene's	0,168595
	V+G140	Shapiro-Wilk	0,258174	Levene's	0,168595
Suppl 2A CCL5	V-	Shapiro-Wilk	0,138442	Levene's	0,504174
	V+	Shapiro-Wilk	0,874697	Levene's	0,504174
	V+G140	Shapiro-Wilk	0,94073	Levene's	0,504174
Suppl 2A TNF	V-	Shapiro-Wilk	0,53944	Levene's	0,539249
	V+	Shapiro-Wilk	0,825863	Levene's	0,539249
	V+G140	Shapiro-Wilk	0,971188	Levene's	0,539249
Suppl 2A IFNB	V-	Shapiro-Wilk	0,380092	Levene's	0,825863
	V+	Shapiro-Wilk	0,438929	Levene's	0,825863

	V+G140	Shapiro-Wilk	0,336844	Levene’s	0,825863
Suppl 2B CCL2	dsDNA	Shapiro-Wilk	0,918794	Levene’s	0,710189
	dsDNA +G140	Shapiro-Wilk	0,179161	Levene’s	0,710189
Suppl 2B CCL5	dsDNA	Shapiro-Wilk	0,23366	Levene’s	0,367216
	dsDNA +G140	Shapiro-Wilk	0,69518	Levene’s	0,367216
Suppl 2B TNF	dsDNA	Shapiro-Wilk	0,740016	Levene’s	0,889708
	dsDNA +G140	Shapiro-Wilk	0,088267	Levene’s	0,889708
Suppl 2B IFNB	dsDNA	Shapiro-Wilk	0,945669	Levene’s	0,496458
	dsDNA +G140	Shapiro-Wilk	0,126851	Levene’s	0,496458
Suppl 2C P21	V-	Shapiro-Wilk	0,347335	Levene’s	0,40142
	V+	Shapiro-Wilk	0,835828	Levene’s	0,40142
Suppl 3A pNFkB Astrocytes	Poly I:C	Shapiro-Wilk	0.100709	Levene’s	0.383328
	V+	Shapiro-Wilk	0.640041	Levene’s	0.383328
	V+G140	Shapiro-Wilk	0.600036	Levene’s	0.383328
Suppl 3B pNFkB Microglia	Poly I:C	Shapiro-Wilk	0.905282	Levene’s	0.82576
	V+	Shapiro-Wilk	0.529202	Levene’s	0.82576
	V+G140	Shapiro-Wilk	0.208861	Levene’s	0.82576
Suppl 3A NFkB Astrocytes	Poly I:C	Shapiro-Wilk	0.015537	Levene’s	0.645336
	V+	Shapiro-Wilk	0.464293	Levene’s	0.645336
	V+G140	Shapiro-Wilk	0.103926	Levene’s	0.645336
Suppl 3B NFkB Microglia	Poly I:C	Shapiro-Wilk	0.614237	Levene’s	0.50631
	V+	Shapiro-Wilk	0.16935	Levene’s	0.50631
	V+G140	Shapiro-Wilk	0.974147	Levene’s	0.50631
Suppl 3C pTBK1 Astrocytes	Poly I:C	Shapiro-Wilk	0.820612	Levene’s	0.181056
	V+	Shapiro-Wilk	0.733119	Levene’s	0.181056
	V+G140	Shapiro-Wilk	0.063692	Levene’s	0.181056
Suppl 3D pTBK1 Microglia	Poly I:C	Shapiro-Wilk	0.160427	Levene’s	0.349436
	V+	Shapiro-Wilk	0.723734	Levene’s	0.349436
	V+G140	Shapiro-Wilk	0.237104	Levene’s	0.349436
Suppl 3C TBK1 Astrocytes	Poly I:C	Shapiro-Wilk	0.146419	Levene’s	0.494703
	V+	Shapiro-Wilk	0.484001	Levene’s	0.494703
	V+G140	Shapiro-Wilk	0.08428	Levene’s	0.494703
Suppl 3D TBK1 Microglia	Poly I:C	Shapiro-Wilk	0.6533	Levene’s	0.429317
	V+	Shapiro-Wilk	0.87091	Levene’s	0.429317
	V+G140	Shapiro-Wilk	0.875381	Levene’s	0.429317
Suppl 4A Synapsin 1	ND	ND	ND	ND	ND
Suppl 4B Burst Duration	Bi	Anderson-Darling	0.00005	Levene’s	3.82 x 10^-5
	3h	Anderson-Darling	0.00005	Levene’s	3.82 x 10^-5
	6h	Anderson-Darling	0.00005	Levene’s	3.82 x 10^-5
Suppl 4C Number of Bursts	Bi	Shapiro-Wilk	0.5722	Levene’s	0.8521
	3h	Shapiro-Wilk	0.6312	Levene’s	0.8521
	6h	Shapiro-Wilk	0.3073	Levene’s	0.8521
	24h	Shapiro-Wilk	0.1319	Levene’s	0.8521
